# Sepsis pathogenesis and outcome are shaped by the balance between the transcriptional states of systemic inflammation and antimicrobial response

**DOI:** 10.1016/j.xcrm.2024.101829

**Published:** 2024-11-19

**Authors:** Rachel Brandes-Leibovitz, Anca Riza, Gal Yankovitz, Andrei Pirvu, Stefania Dorobantu, Adina Dragos, Ioana Streata, Isis Ricaño-Ponce, Aline de Nooijer, Florentina Dumitrescu, Nikolaos Antonakos, Eleni Antoniadou, George Dimopoulos, Ioannis Koutsodimitropoulos, Theano Kontopoulou, Dimitra Markopoulou, Eleni Aimoniotou, Apostolos Komnos, George N. Dalekos, Mihai Ioana, Evangelos J. Giamarellos-Bourboulis, Irit Gat-Viks, Mihai G. Netea

**Affiliations:** 1 The Shmunis School of Biomedicine and Cancer Research, George S. Wise Faculty of Life Sciences, Tel Aviv University, Tel Aviv, Israel; 2 Human Genomics Laboratory, University of Medicine and Pharmacy of Craiova, Craiova, Romania; 3 Regional Centre of Medical Genetics Dolj, County Clinical Emergency Hospital Craiova, Craiova, Romania; 4 Hospital for Infectious Diseases and Pneumology "Victor Babeş" Craiova, Craiova, Romania; 5 Infectious Disease Department, University of Medicine and Pharmacy of Craiova, Craiova, Romania; 6 4^th^ Department of Internal Medicine, National and Kapodistrian University of Athens, Medical School, Athens, Greece; 7 Intensive Care Unit, G. Gennimatas Thessaloniki General Hospital, Thessaloniki, Greece; 8 3^rd^ Department of Critical Care Medicine, National and Kapodistrian University of Athens, Medical School, Athens, Greece; 9 Intensive Care Unit, Latseion General Hospital, Elefsis, Greece; 10 1^st^ Department of Internal Medicine, Evangelismos Athens General Hospital, Athens, Greece; 11 Intensive Care Unit, KAT General Hospital, Kifissia, Athens, Greece; 12 Intensive Care Unit, Aghios Dimitrios General Hospital, Thessaloniki, Greece; 13 Intensive Care Unit, Koutlibaneion and Triantafylleion General Hospital, Larissa, Greece; 14 Department of Medicine and Research Laboratory of Internal Medicine, National Expertise Center of Greece in Autoimmune Liver Diseases, European Reference Network on Hepatological Diseases (ERN RARE-LIVER), General University Hospital of Larissa, Larissa, Greece; 15 Hellenic Institute for the Study of Sepsis, Athens, Greece; 16 Department of Internal Medicine and Radboud Center for Infectious Diseases, Radboud University Medical Center, Nijmegen, the Netherlands; 17 Department of Immunology and Metabolism, Life & Medical Sciences Institute, University of Bonn, Bonn, Germany

**Keywords:** sepsis, septic shock, infection, immune response, systemic inflammation, patient stratification, personalized medicine, immunotheraphy, precision medicine

## Abstract

Patients with sepsis differ in their clinical presentations and immune dysregulation in response to infection, but the fundamental processes that determine this heterogeneity remain elusive. Here, we aim to understand which types of immune dysregulation characterize patients with sepsis. To that end, we investigate sepsis pathogenesis in the context of two transcriptional states: one represents the immune response to eliminate pathogens (resistance, R) and the other is associated with systemic inflammation (SI). We find that patients with sepsis share a molecular fingerprint of a low R-to-SI balance—i.e., a low R relative to the level of SI. Differences between patients with sepsis are explained by the wide diversity of R and SI states that fall under this fingerprint, such as patients with high SI, patients with low R, or both. We show how this R/SI framework can be used to guide patient stratification that is relevant to disease prognosis and management, outperforming existing classifications of sepsis.

## Highlights

• Transcriptional programs of “resistance” and “systemic inflammation” are described
• Sepsis is characterized by a low “resistance-to-systemic inflammation” balance
• This framework can stratify patients with sepsis for prognosis and management

## Graphical abstract

**Figure:**
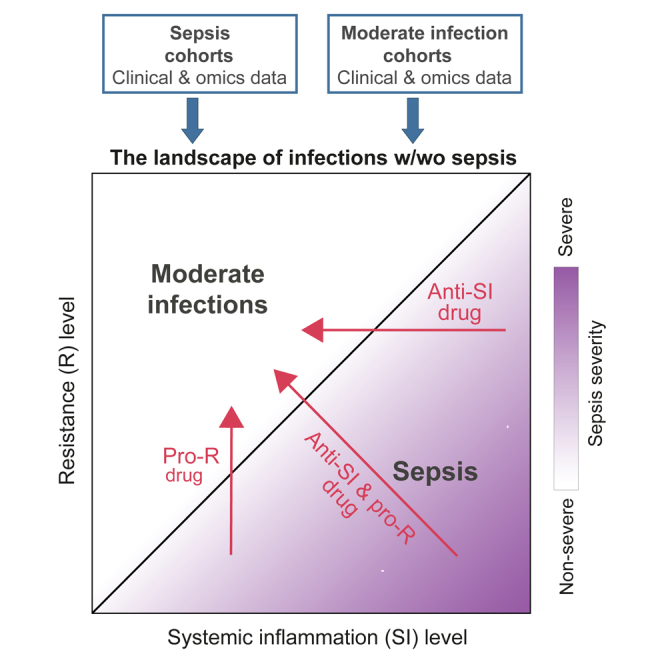

## Teaser

Brandes-Leibovitz et al. propose a framework for patient stratification in sepsis based on transcriptional programs characterizing functional states of “resistance” (R) and “systemic inflammation” (SI). Sepsis is characterized by a low R-to-SI balance, and patient stratification based on this functional approach outperforms current classifications for better patient prognosis and management.

## Introduction

Sepsis is a pathological condition resulting from dysregulated immune responses in patients with infections, leading to severe symptomatology, organ dysfunction, and often death.^1^ Sepsis is one of the most important causes of morbidity and mortality in both developed and developing countries, with estimated 49 million cases and 11 million deaths globally every year.^2^ Antibiotics and intensive care units have significantly decreased sepsis mortality during the 20^th^ century, but the outcome of sepsis remained largely stable in the last two decades.^2^ It has been hypothesized that immunotherapy will be the next revolution in the treatment of sepsis, yet this has never materialized despite a plethora of clinical trials with anti-inflammatory immunotherapies that have all failed to improve the outcome of the patients. It is believed that the heterogeneity of sepsis at the level of causal microorganism, source of infection, and especially the type of immune dysregulation has led to the failure of sepsis immunotherapy trials.^3^ Indeed, some patients with sepsis display hyperinflammatory characteristics (the so-called *macrophage activation-like syndrome*, MALS^4^), other patients show interruption of critical immune functions (*immunosuppression*^5^^,^^6^^,^^7^^,^^8^), and in some critically ill patients with sepsis, these two types of immune dysregulation can occur at the same time or change in time depending on the phase of disease. Understanding the heterogeneity of immune dysregulation in sepsis is necessary for the development of better therapeutic approaches.

Recent studies have characterized sepsis subtypes based on whole-blood transcriptomes, primarily relying on systematic analyses within patients with sepsis.^3^^,^^9^^,^^10^^,^^11^^,^^12^^,^^13^^,^^14^^,^^15^^,^^16^^,^^17^^,^^18^^,^^19^^,^^20^^,^^21^ However, there are several challenges for the identification of immune subtypes in sepsis. First, there is little reproducibility (overlap) of subtypes across different sepsis studies, limiting the ability to identify shared targets for immunomodulation.^22^ Second, there is a lack of consistency between current datasets (e.g., datasets differ in how sepsis is defined, infection type, and age group) and it is challenging to integrate them. In accordance, most studies do not integrate information from multiple cohorts. Finally, differences between sepsis and infections without sepsis are not commonly exploited in the analysis of sepsis, and current datasets are limited to small cohorts and a particular type of infection.^23^

Given these challenges, much remains unknown about immune dysregulation in patients with sepsis. First, which types of immune dysregulation characterize patients with sepsis is not fully described. Second, it is not clear how to identify the different types of immune dysregulation. Finally, many of the key pathophysiological pathways that can be immunotherapeutically targeted in sepsis remain unknown.

To address these goals, we constructed a model of immune dysregulation in sepsis by combining transcriptomes from different cohorts of sepsis in combination with various cohorts of infections without sepsis (termed “moderate infections”). This approach was designed to improve reproducibility and enable the identification of sepsis-specific dysregulations. To integrate these datasets, we exploited predefined transcriptional programs of inflammation and host defense recently described in an experimental model. These programs are based on extensive assessments of both pathogen persistence and tissue inflammation, a transcriptional signature of immune activation that is associated with the detection and elimination of the invading pathogen (resistance [R]).^24^ In addition, we have recently used extensive assessment of immunological and clinical measures to describe a transcriptional signature that is associated with the physiological status of systemic inflammation (systemic inflammation [SI]).^25^ Here, we show that these two programs are valuable for a systematic analysis of moderate infections and sepsis and then investigate a compendium of sepsis and moderate infection cohorts in the context of these programs.

Several key findings emerge from this analysis. First, we found that the transcriptional states of a low R program and a high SI program, and particularly a low R-to-SI ratio, are a general fingerprint of sepsis: it is reproducible across multiple sepsis cohorts, and it can be used to distinguish sepsis from infections without sepsis. Second, we show that the relative R-to-SI states are associated with sepsis severity and mortality. Thus, the results establish two key types of molecular dysregulation of sepsis, a low R and a high SI, whose balance is linked to sepsis, sepsis severity, and mortality. We demonstrate that these two types of immune dysregulation should be approached differently from a therapeutic point of view, we propose ways to identify them, and we identify pathways that are associated with them. Finally, we validated the usefulness of the R/SI states for the stratification of patients into endotypes, outperforming existing classifications of sepsis.

## Results

### Characterization of the R and SI programs in moderate infection and sepsis

To identify predefined transcriptional immune programs that could define immune dysregulation in sepsis, we constructed an unbiased set of 76 candidate immune programs and systematically benchmarked each of these programs using two criteria: a high covariation during infections in general, and during sepsis in particular. The analysis identified two best-performing programs: first, a “resistance” program, with high covariation during infections as well as specificity to infections, and second, the “systemic inflammation” program, with the best covariation during sepsis (Figure S1A and S1B, STAR Methods). Given that programs R and SI were originally identified in another context (R – influenza A virus [IAV] infection in mice,^24^ SI – chronic systemic inflammation in humans^25^), in the following studies, we confirmed the general relevance of these two programs in human blood samples, during moderate infections and sepsis.

Several lines of evidence confirmed the relevance of R and SI in moderate infections. First, analysis of inter-individual and inter-gene variation indicates that both R and SI are valid for the study of human blood in healthy subjects, and during both bacterial and viral infections (Figures S1C–S1E). Second, both SI- and R-associated genes are induced during viral and bacterial infections (Figure S1A; Table S1). We note that the R and SI were confirmed as distinct programs: each program has significant contribution to the variation during infection (Figures S1C–S1E),^24^^,^^25^ the two programs are linked to a distinct inflammatory plasma state (Figure S1F),^24^^,^^25^ and only SI (but not R) is responding in SI with negative blood culture (Figures S1A and S1G).

**Figure 1:**
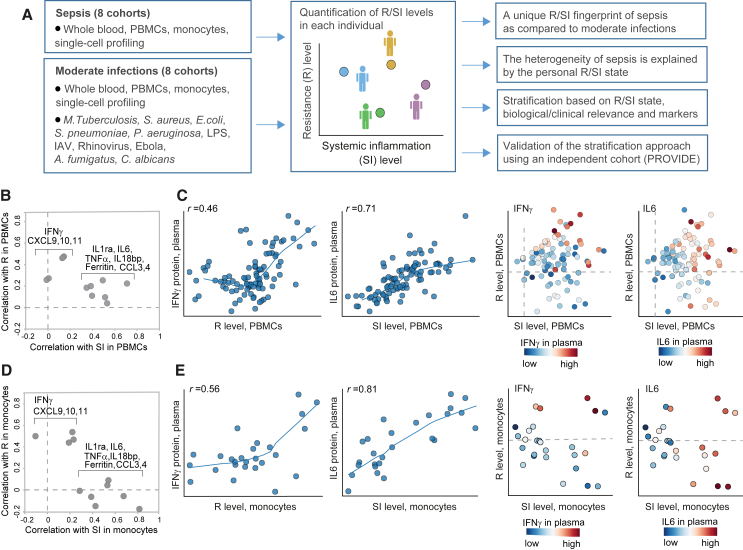
Global transcriptional states of systemic inflammation and resistance in patients with sepsis (A) Schematic of methodology: integrative analysis of sepsis and moderate infections, including the PBMCs/monocytes data from the FUSE cohort. Personal levels of resistance (R) and systemic inflammation (SI) were calculated for each subject. (B) R and SI are associated with two distinct inflammatory states in sepsis. The scatterplot compares, for each protein (a dot), its correlation with SI levels (x axis) and its correlation with R levels (y axis). Correlations (*r*) were calculated across patients with sepsis from the FUSE cohort; R and SI levels were calculated using expression profiles in PBMCs. Included are selected pro-inflammatory plasma protein markers. IL-6 and IFNγ are exemplified in C. Findings are consistent with previous studies (Figure S1F). (C) Associations of the plasma IFNγ and IL-6 proteins with R and SI levels in PBMCs of patients with sepsis. Left: scatterplots for R or SI levels (x axis) against protein abundance (y axis) across individuals with sepsis (dots). Right: scatterplot for the SI and R levels (x and y axis, respectively) of each patient with sepsis (a dot), where each patient is colored by its plasma level of a certain protein (indicated on top). R and SI levels were calculated using the expression profiles in PBMCs from the FUSE cohort. (D and E) Validation in monocytes. Plots D and E are shown as in plots B and C, respectively, but for R/SI levels that were calculated using expression profiles of blood-derived monocytes (rather than PBMCs). Data of patients with sepsis from the FUSE cohort. Related to Figure S1.

Next, we confirmed the relevance of programs R and SI in sepsis. To that end, we measured transcriptomes of peripheral blood mononuclear cells (PBMCs) derived from the blood of patients with sepsis (*N* = 125) and healthy control subjects (*N* = 284) (FUSE cohort,^26^ STAR Methods) and quantified the R and SI levels of each subject based on its measured transcriptome. Several lines of evidence support the validity of the inferred R and SI levels in PBMCs of patients with sepsis. First, using measurements of plasma proteins in the FUSE cohort, we confirmed that the induction of both R and SI is positively correlated with a variety of immune activation markers, consistent with the notion that each of these two programs is part of the host immune response. Second, in line with previous reports,^24^^,^^25^ R and SI differed in their associations to plasma biomarkers: the plasma concentrations of IFNγ, CXCL10, and CXCL11 proteins in sepsis were mainly correlated with R levels of PBMCs (Pearson’s *r* = 0.46, 0.47, 0.21, *p* < 10^−5^, 10^−5^, 0.07, respectively), whereas plasma concentrations of IL-6 and IL-18bp were mainly correlated with SI levels of PBMCs (Pearson’s *r* = 0.71, 0.34, *p* < 10^−15^, 10^−3^) (cf. Figures 1B and S1F). Finally, R and SI levels explain a large fraction of the global transcriptional response in sepsis (Figures S1C–S1E), validating the applicability of our approach.

In addition, we tested the relevance of R and SI in monocytes from patients with sepsis, based on the assumption that much of the R/SI signal in PBMCs stems for the molecular states of monocytes, as crucial cell type in sepsis. To test this, we measured the transcriptomes of isolated monocytes derived from the blood of 36 sepsis and 15 healthy subjects from the FUSE cohort (STAR Methods); each transcription profile was subsequently used to quantify the R and SI levels of monocytes in an individual subject. Indeed, the associations of plasma proteins with R and SI were similar in monocytes and PBMCs (Figures 1B and 1C versus 1D and 1E), supporting the hypothesis that the R/SI levels of PBMCs are consistent with R/SI levels of monocytes. Additionally, the monocytes’ R and SI levels explain a significant fraction of the individual variation in thousands of genes (Figures S1C–S1E), indicating that R and SI are two global programs that together govern transcriptional states of monocytes during sepsis. We therefore focused on the R and SI programs to investigate sepsis throughout this study.

### A dysbalance between the states of R and SI is a fingerprint of sepsis

To investigate sepsis and moderate infections in an unbiased manner, we combined the transcriptome FUSE data with additional datasets of varying patient and sample characteristics, including three sepsis and septic shock datasets of gene expression in blood,^9^^,^^10^^,^^11^ and several independent datasets of moderate (non-sepsis) infection: IAV infection,^27^ *M. tuberculosis*^28^ and *S. aureus* infection,^29^ *ex vivo* stimulations of human PBMCs with *A. fumigatus*, *C. albicans*, *M. tuberculosis*, *P. aeruginosa*, and *S. pneumoniae* infections,^30^ and an *ex vivo* lipopolysaccharide (LPS) stimulation of murine macrophages^31^ (datasets #1–#10 in Table S2 and in STAR Methods).

**Figure 2:**
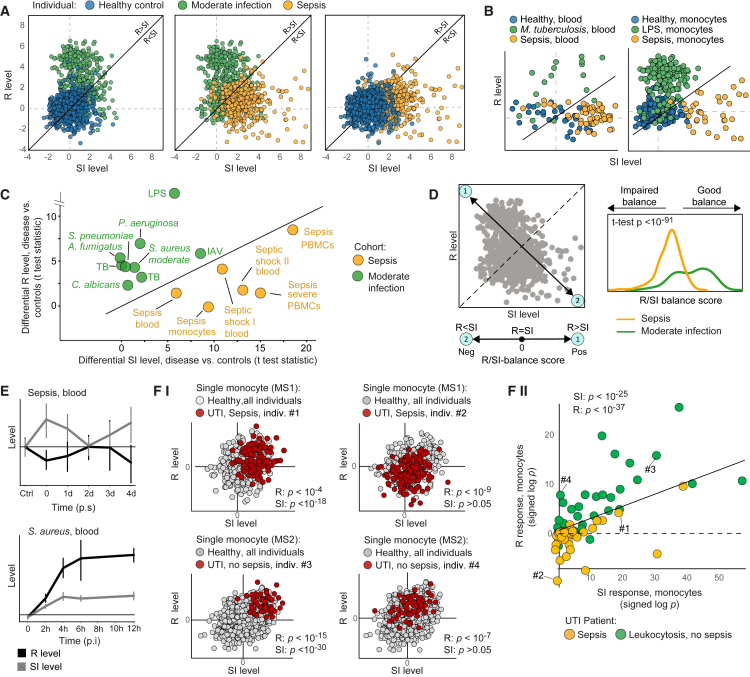
Sepsis is marked by a dysbalanced cell state of low R relative to the SI level In (A)–(D), data from multiple independent cohorts of either blood, PBMCs, or monocyte profiling (datasets #1–#10 in STAR Methods). (A and B) The levels of SI (x axis) and R (y axis) across individuals (dots) from all cohorts (A) or specific cohorts (B). (C) Differential R and SI levels (disease versus controls, standard t test statistics) across cohorts (dots). Abbreviations: TB, *M. tuberculosis*. (D) The “R/SI-balance score” is a biomarker of sepsis. Left: the R/SI-balance score is defined as R minus SI—that is, the score is decreasing along the top-left-to-bottom-right diagonal, where positive and negative scores indicate R > SI and R < SI, respectively. Right: the distributions of individuals by their R/SI-balance scores, revealing lower balance scores in sepsis (an “impaired” R/SI balance) compared to moderate infections (a “good” R/SI balance). (E) R and SI levels across time points during infection. Included are time-series dataset (datasets #12 and #13 in STAR Methods). Error bars: 95% confidence intervals. p.i., post infection; p.s., post symptoms. Related to Figure S2. (F) R and SI responses to uronary tract infection (UTI) at single-cell resolution (dataset #17 in STAR Methods). (F–I) For each single monocyte (a dot), the plot presents its R and SI levels; each plot presents specific monocyte subpopulation (MS1 or MS2) for all controls (gray) or one patient (red). Presented are *p* values (t test) for the bias in single-cell R/SI levels in one patient versus all controls (for a given monocyte subpopulation). These *p* values (log_10_-scaled and signed by direction) are referred to as the *R* (*or SI*) *response*. (F-II) Each dot provides the R and SI responses for a single patient and a certain monocyte subpopulation. R/SI responses of individuals #1–#4 (indicated in plot F-II) are exemplified in plot F–I. Patients with sepsis (yellow) and moderate infection (green) UTI are included. *p* values (indicated on top) are detailed in Figure S3.

In a systematic dissection of the R and SI states across all individuals from all datasets, we found a sepsis-specific signature of R and SI. In individuals with infections of low or moderate severity, both R and SI levels are elevated, but R tends to be activated at higher levels compared to the SI levels. However, this response is hampered in patients with sepsis, such that R is low relatively to the SI (Figure 2A). To further demonstrate the relevance of our finding, we analyzed each dataset independently. In datasets of moderate inflammatory conditions, we found that the induction of R is similar or higher than the induction of SI levels, whereas in sepsis, the induction of R is low relative to the induction of SI (Figures 2B, 2C, S2A, and S2B). As a quantitative assessment for the relative levels of R and SI, we subtracted the SI level from the R level (the “R/SI-balance score,” Figure 2D, left). Indeed, we observed a significant separation between sepsis and moderate infections by their R/SI-balance scores (t test *p* < 10^−86^, Figure 2D, right). We conclude that the low level of R relatively to the level of SI is common to the various endotypes of sepsis: patients with moderate infection more likely have R levels that are higher than the SI levels (a high, or a “good”, R/SI balance), whereas patients with sepsis more likely have R levels that are lower than their SI levels (a low, or an “impaired,” R/SI balance).

We next asked whether the impaired R/SI balance is sustained during the progression of sepsis or whether it appears transiently during the course of disease. To address this, we analyzed time-series datasets of infections^32^^,^^33^^,^^34^ (Table S2, STAR Methods). In human sepsis, we found that the signature of low R relative to SI is sustained during five days after the clinical recognition of severe disease (Figure 2E), supporting our general findings regarding the low R/SI balance in sepsis. This signature is not apparent during the course of moderate infections (Figures 2E and S2C), as expected. A murine model of sepsis (*in vivo* LPS stimulation^35^) further allowed us to study the trajectory of response before the clinical recognition of severe sepsis. In PBMCs, we found that the R program peaks at early time points (6–12 h) post stimulation and is already repressed at 5 days post stimulation; the SI response, in contrast, peaks in intermediate time points (2–3 days post stimulation) and is still high at 5 days post stimulation (Figure S2D). Together, these differences in temporal dynamics lead to a very low R/SI balance at 5 days post stimulation. Interestingly, similar results were obtained in additional tissues, such as bone marrow and spleen (Figure S2E). We conclude that sepsis is characterized by a transition from a trajectory within the region of high R/SI balance toward the region of low R/SI balance.

### Sepsis is marked by a dysbalance between R and SI at the molecular level

Given the impaired R/SI balance in bulk samples, we tested the R and SI at the resolution of single cells. Particularly, we analyzed single-cell RNA sequencing data of urinary tract infection (UTI)^23^ (STAR Methods). We first focused on monocyte subpopulations. After calculation of R and SI level in each single monocyte separately (exemplified in Figure S3A), we found that single-cell R and SI levels are highly consistent in each individual patient—for instance, monocytes of subpopulation MS1 in patient #2 with sepsis tend to low R levels compared to MS1 monocytes of healthy controls (*p* < 10^−9^, t test; Figure 2F–2I). We used the *p* value of this t test as a score for the molecular level of R (or SI) in each patient compared to controls and refer to this score as the “*R* (*or SI*) *response*” (signed log_10_ *p* value, positive/negative scores for increasing/decreasing levels, exemplified for individuals #1–#4 in Figure 2F–I, II). Overall, for 80% of the patients, the monocytes demonstrate significant (*p* < 0.05) molecular responses of either R or SI (Figure S3B). These findings suggest a cell-intrinsic R and SI state in monocytes. To further compare sepsis and moderate infection, we subdivided the patients with UTI into two groups: 10 patients with UTI that had a clear and persistent organ dysfunction (sepsis) and 10 patients that had leukocytosis but no organ dysfunction (moderate infection). We found that the response of single monocytes from sepsis and moderate infection differed, showing a bias of patients with sepsis toward a lower R response while retaining a high SI response (Figures 2F–II, and S3C). Thus, sepsis monocytes are characterized by an impaired R/SI balance at the molecular level.

Next, the analysis of monocytes (Figure 2F) was also applied to three types of lymphocytes (T, B, and natural killer [NK] cells) using the same dataset. We found that T, B, and NK cells demonstrate significant (*p* < 0.05) molecular responses of either R or SI in 63%, 43%, and 38% of the patients, respectively (Figures S3D–S3F), supporting the notion of a cell-intrinsic R and SI levels in various types of lymphocytes. Furthermore, sepsis is characterized by reduced-R/elevated-SI (compared to moderate infection) in both T cells and B cells, as observed in monocytes (Figure S3C). Thus, our findings suggest an impaired R/SI balance at the molecular level in several innate and adaptive immune cell types.

### The dysbalance between R and SI is linked to the heterogeneity of sepsis phenotypes

Given that sepsis is marked by the cell state of an impaired R/SI balance, we next asked whether and how the R/SI states are related to the heterogeneity of sepsis. We focused on the heterogeneity in various pathophysiological measures, including (1) plasma protein concentrations, (2) gene expression in immune cells, and (3) clinical phenotypes. For each category, we show that the pathophysiological heterogeneity within sepsis is associated with the state of an impaired R/SI balance.

**Figure 3:**
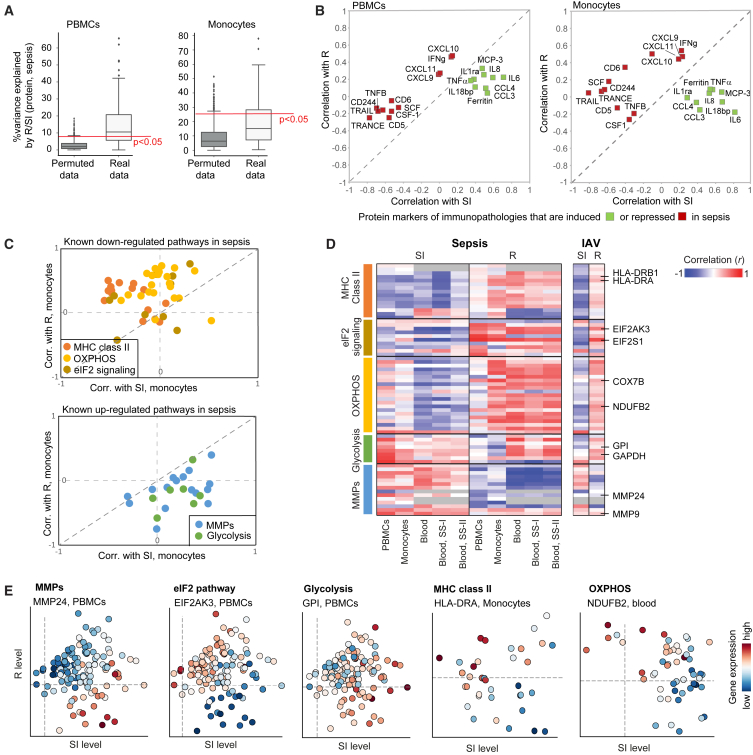
The heterogeneity of genes and plasma proteins in sepsis is associated with the impaired R/SI state Phenotypic variation in sepsis, either in plasma protein concentrations (A and B) or in PBMCs/monocytes mRNA levels (C–E), is associated with the low R and high SI cell state. Measurements of monocytes and PBMCs are from the FUSE dataset. (A) Boxplots for the percentage of inter-individual variance in proteins that is explained by a linear combination of R and SI, using either real (white) or permuted (gray) data. R/SI levels were calculated either using transcriptomes from PBMCs (left) or monocytes (right). (B) Protein markers of immunopathology in sepsis are associated with the impaired R/SI balance. The scatterplot compares, for each protein marker of immunopathology (a dot), its correlation with SI levels (x axis) and R levels (y axis) across patients with sepsis. R and SI levels were calculated using expression profiles in either PBMCs (left) or monocytes (right). Included are markers for immune dysfunctions that have a known up- or down-regulation in sepsis (color coded). (C–E) Analysis of previously reported pathways that are up- or down-regulated in sepsis. (C) The scatterplots compare, for the expression of each gene (a dot), its correlation with SI levels (x axis) and R levels (y axis). Correlations were calculated using data in monocytes across patients with sepsis. (D) Shown are correlations (color-coded) between each gene (a row) and the SI or R levels (columns), calculated based on transcriptomes in each cohort (columns; datasets #1–#5 in STAR Methods). Abbreviations: SS-I/II, septic shock I and II. (E) Examples of selected genes from D, as shown in Figures 1C and 1E. Genes are indicated on top. Related to Figure S4.

(1) *Plasma proteins.* Using plasma proteomics in sepsis (the FUSE cohort^26^), we could quantify the fraction of inter-individual variation in protein concentrations that is explained by the R and SI levels (Table S3). For 62 of the 97 measured proteins, the combination of PBMCs’ R and SI explained significant fractions of the variation (empirical *p* < 0.05; Figure 3A, left). Monocytes’ R and SI levels also explained substantial fractions of the variation (27 of 97 proteins with empirical *p* < 0.05; Figure 3A, right). Focusing on established protein markers of immune malfunctions in sepsis, we distinguished between markers of immune functions that are increased in sepsis (such as ferritin, IL-6, IL-18bp, and IL-8 as inflammation markers) and markers of immune functions that are repressed in sepsis (such as CD5, CD6 and CD244, SCF [lymphopoiesis], IFNγ and IFNγ-inducible cytokines [immune activation], CSF-1 [proliferation and phagocytosis], and TRANCE [an inhibitor of apoptosis]). As shown in Figure 3B, biomarkers that are increased had stronger correlations with increasing SI levels compared to the correlations with R levels; in contrast, markers of repressed functions showed stronger correlations with increasing R levels compared to the correlations with SI (with similar findings in monocytes and PBMCs). Similar findings were obtained when accounting for confounders (Figure S4A). Thus, plasma proteome is associated with the impaired R/SI balance in PBMCs and monocytes.

Of note, we cannot determine causality—for instance, whether plasma proteins affect monocytes, are affected by monocytes, or other cell types affect both the plasma proteins and monocytes. However, the observed associations suggest the ability of plasma proteins to mediate between cellular R/SI states of different cell types. Indeed, we found that the composition of the septic plasma contributes to the impaired R/SI balance of various cell types (Figure S4B). Thus, complex interactions involving multiple cell types and plasma proteins are likely to contribute to the impaired R/SI balance in sepsis.

**Figure 4:**
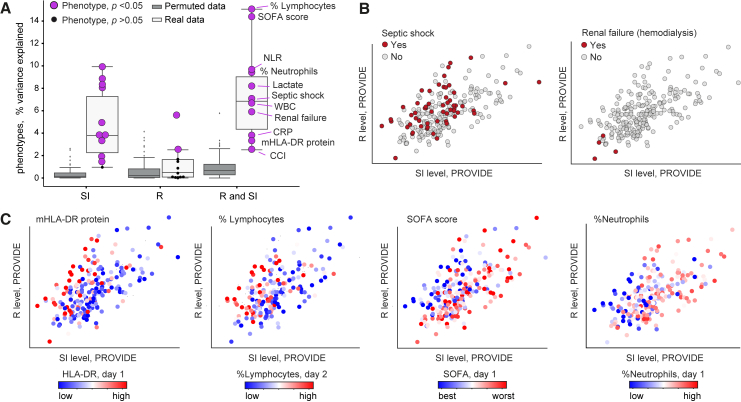
The heterogeneity of clinical parameters within sepsis is associated with the impaired R/SI cell state Data are shown for several physiological phenotypes across the PROVIDE clinical trial: SOFA scores in day 1 of hospitalization, quantity of the mHLA-DR protein in day 1 of hospitalization, the percentage of circulatory lymphocytes in day 2 of hospitalization, as well as septic shock, CCI, percentage of neutrophils, NLR, WBCs, lactate, CRP, and renal failure in day 1 of hospitalization. (A) Boxplots for the percentages of inter-individual variance in phenotypes that are explained by either SI (left), R (middle), or the linear combination of R and SI (right), using either real (white) and permuted (gray) data. Each phenotype (a dot) is colored by purple/black for a significant/insignificant (empirical *p* < 0.05) percentage of explained variation. (B and C) Scatterplots for SI and R levels (x and y axis, respectively) of each patient with sepsis (a dot), where each patient is colored by its level of a certain clinical parameter (indicated on top). The plots demonstrate the utility of the R and SI levels as biomarkers for the pathophysiology of sepsis. Related to Figure S5.

(1) *Gene expression.* As noted earlier, the R and SI levels explain substantial fraction of the global gene expression variation during infection (Figures S1C–S1E), supporting the notion that the quantification of R and SI reflects the global transcription profile rather than one or a few specific pathways. Encouraged by this observation, we examined whether known transcriptional hallmarks of sepsis are associated with the impaired R/SI balance. To test this, we used established mRNA markers of pathological molecular states in sepsis, including the upregulation of genes involved in glycolysis and matrix metalloproteinases (MMPs) and downregulation of major histocompatibility complex (MHC) class II, eIF2 signaling, and oxidative phosphorylation (OXPHOS) genes.^6^ The impaired R/SI balance is indeed consistent with the established transcriptional changes in sepsis: the sepsis-upregulated functions have stronger correlations with increasing SI levels compared to the correlations with R levels (glycolysis, MMPs; e.g., GPI and MMP24), whereas the sepsis-downregulated functions demonstrate the opposite trend (eIF2 signaling, OXPHOS, and MHC class II, e.g., EIF2AK3, NDUFB2, and HLA-DRA) (Figures 3C–3E, S4C, and S4D). Thus, both induced and repressed functions in sepsis are linked to the impaired R/SI balance. Notably, the same findings were found in both sepsis and moderate infections (Figures 3D and 3E), suggesting that many sepsis-related dysfunctions are a consequence of the global R/SI imbalance rather than a consequence of specific defects of sepsis.
(2) *Clinical phenotypes.* To assess whether the R and SI states are related to the wide phenotypic diversity in sepsis, we analyzed an independent dataset—the PROVIDE clinical trial^21^—consisting of 223 patients with sepsis with high mortality (59% 28-day mortality, Table S2; Figures S5A and S5B). Several phenotypes were assessed, such as the SOFA score, mHLA-DR protein, white blood cells (WBCs), neutrophil-to-lymphocyte ratio (NLR), lymphopoiesis, renal failure, and the R/SI levels (STAR Methods). For all clinical tests, either R, SI, or their combination explained significant fractions of the phenotypic diversity (empirical *p* < 0.05, Figure 4A). The pathologies of sepsis were associated with either high SI, low R, or both (Figures 4B, 4C, S5C, and S5D). We obtained similar results using a predefined measure of severity in the FUSE cohort (Figure S5E), and the R/SI-balance score outperformed alternative markers in its association with disease severity (Figures S5D-II and S5E-IV). As additional support, we observed that the R/SI-balance score also presents a strong prognostic capacity for survival at 28 days (p < 0.0002, Cox proportional hazards [CPH] model predicting 28-day mortality using the R/SI balance as a continuous variable with additional parameters of age and gender; Table S4). Overall, the impaired R/SI balance is associated with sepsis, sepsis severity, and mortality.

### Potential implications of the R/SI framework

The aforementioned analysis established two types of immune dysregulation of sepsis, low R and high SI, making several unanticipated insights.

**Figure 5:**
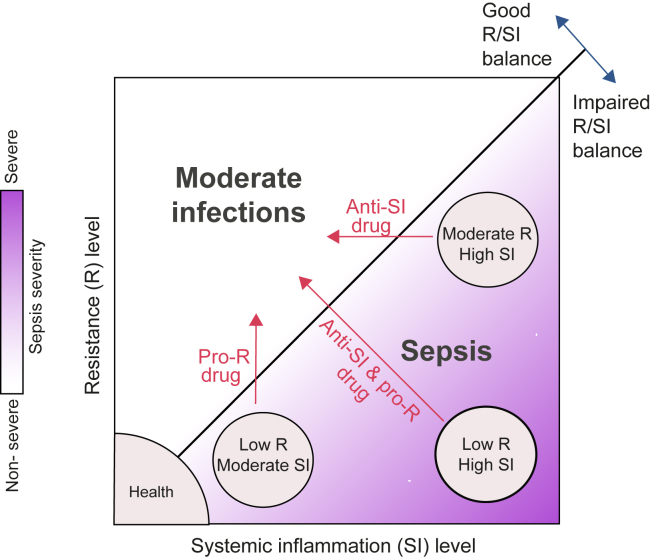
A model of sepsis based on the molecular states of R and SI The molecular state of a low R-to-SI balance is a fingerprint of sepsis, as opposed to infections of moderate severity. The heterogeneity in sepsis is explained by the wide diversity of R/SI levels that fall under the broad fingerprint of sepsis (e.g., patients with high SI, patients of severe R/SI imbalance). The model suggests that each patient with sepsis should be treated either with a pro-R drug, an anti-SI drug, or both.

First, our findings imply that the two identified types of immune dysfunctions should be approached differently from a therapeutic point of view. In particular, each patient should be treated either with a pro-R drug, an anti-SI drug, or both—depending on the specific combination of R and SI levels (Figure 5). In accordance, the model predicts that R and SI could be modified together (increasing R and decreasing SI) or independently.

Second, as R and SI are transcriptional states, it is possible to test the effect of therapeutic interventions on the R/SI balance in an *ex vivo* setting. To demonstrate this, we analyzed the effect of several drugs on monocytes (data from Hu et al.^36^). We found an effect (induction) of IFNγ only on the R program, but not on the SI program (Figure S5F), supporting the notion that each program can be modulated independently. Additional examination of the states arising from anti-inflammatory modulators revealed that in many cases both R and SI levels are inhibited (Figure S5F). As the anti-SI effect is generally beneficial but the anti-R effect is likely detrimental in sepsis, this observation highlights the importance of a rationale selection of drugs based on a joint analysis of both R and SI—searching for a drug that has a specific suppressive effect only on program SI and/or an inducing effect only on program R.

Finally, the framework allowed us to identify molecular pathways that are associated with the low-R/high-SI state. The results reveal multiple pathways that are associated with the imbalanced state, such as BMP2 signaling, quiescence, extracellular matrix organization, and elastic fiber formation (Figure S6; Table S5).

### Stratification of patients with sepsis using the R/SI model

**Figure 6:**
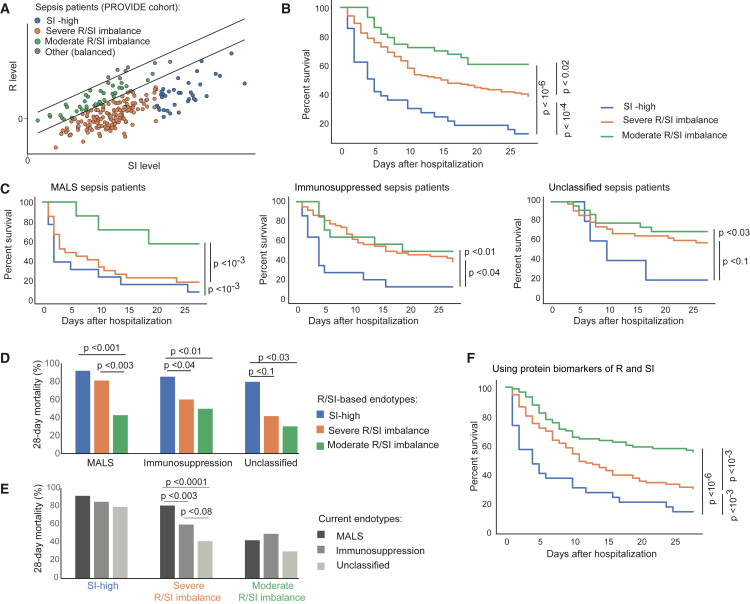
Stratification of patients with sepsis based on their R/SI cell states Analysis of patients with sepsis from the PROVIDE clinical trial.^21^ (A) Three R/SI-based endotypes are indicated. The prognostic capacity of these endotypes is demonstrated in (B)–(F). (B) The prognostic capacity of the R/SI-based endotypes. Kaplan-Meier survival curves for the endotypes (color coded). (C–E) The R/SI-based classification adds prognostic information beyond the current classification. (C) Prognostic capacity of the R/SI-based endotypes within previously defined immune states.^21^^,^^37^^,^^38^ Presented are Kaplan-Meier survival curves for the R/SI-based endotypes. Plots are shown as in B but each plot shows the survival curve within one previously defined endotype (indicated on top). (D) The percentage of 28-day mortality of each R/SI-based endotype (color coded) within previously defined subset of patients (x axis). (E) The percentage of 28-day mortality of each previously defined subset (color coded) within each of the R/SI-based endotypes (x axis). (F) 28-day prognostic capacity of the R/SI-based endotypes when using biomarkers of R and SI. Results are calculated and presented as in B (for the same individuals and endotypes), except from R and SI that were assessed using biomarkers: averaging CXCL11 and IFNγ plasma protein levels for R, and averaging IL-6 and IL-8 plasma protein levels for SI. In B–F, comparison *p* values were calculated using the log rank test and insignificant results (*p* > 0.1) were excluded for simplicity. Related to Figure S5.

We next examined whether R/SI-based endotypes are clinically relevant. To that end, we proposed the following stratification: patients with sepsis with a limited reduction in R/SI balance (“moderate R/SI imbalance”) and patients with low R/SI balance that are subdivided into patients whose SI is exceptionally high (“high-SI”) or not (“severe R/SI imbalance”). We validated these endotypes using the outcome of patients from the PROVIDE cohort (20% moderate R/SI imbalance, 62% severe R/SI imbalance, 16% high-SI; Figure 6A). Table S6 compares the clinical characteristics of these endotypes. As expected, most pathophysiological measures of sepsis differ between the endotypes of moderate and low-grade R/SI imbalance—consistent with the observed link between these measures and the R/SI-balance score (Figure 4). Importantly, each endotype is distinct: the three endotypes differ in their severity of sepsis (SOFA and APACE II scores), circulating lactate concentrations, and WBC count. Apart from the pathophysiology of sepsis, the three endotypes do not significantly differ in other characteristics such as age, gender, comorbidities, type of infection, and administrated antimicrobials.

We further noted that the three endotypes differ in their 28-day survival (Figure 6B): the non-survivors are associated with the SI-high endotype, whereas the survivors are associated with a moderate R/SI balance endotype. Patients with severely decreased R/SI balance are in an intermediate risk class (*p* < 0.02 for difference in time to death with the log rank/Breslow test; Figure 6B). When patients were categorized into the previously defined groups of MALS, immunosuppression, and the remaining unclassified patients,^21^^,^^37^^,^^38^ we found that the R/SI classification presented strong prognostic capacity within each of these groups (Figures 6C and 6D; Table S4)—for instance, the SI-high and moderate-balance endotypes have significant prognostic value within both the patients with MALS (7.7% and 57.2% survival, respectively; *p* < 10^−3^, log-rank test) and the formerly unclassified patients (20% and 69.6% survival, respectively; *p* < 0.03, log rank test; Figures 6D and S5G). In contrast, the previously described classification has limited added prognostic value beyond our proposed classification: a significant contribution only within the severe-R/SI-balance endotype (Figures 6E and S5G). Taken together, results from the PROVIDE cohort argue that the R/SI-based endotypes improved prognostic prediction beyond the current stratification.

We next evaluated whether plasma biomarkers of R and SI can be used for patient stratification. Using the FUSE cohort, the plasma proteins IFNγ and CXCL11 are suggested as top biomarkers of R levels, and IL-6 and IL-8 concentrations are suggested as top biomarkers of SI levels (Figure 3B). These markers were confirmed in the PROVIDE cohort (Figure S5A). We therefore classified the patients into endotypes based on the average concentrations of IFNγ and CXCL11 as R marker, and the average of IL-6 and IL-8 as SI marker. The data showed similar prognostic capacity of both the R/SI levels themselves or the circulating markers of these levels (cf. Figures 6F and 6B; Table S4), demonstrating that it is feasible to identify the R/SI-based endotypes by assessing a limited set of plasma biomarkers. Overall, the R/SI-based stratification is clinically relevant (Figure 6B), outperforms previously described classification (Figures 6C–6E and S5G), and is easy to apply in practice (Figure 6F).

## Discussion

We found that the transcriptional states of two cellular programs, R and SI, capture the complexity of sepsis (Figure 5). (1) The balance between the states of R and SI (namely the “R/SI balance”) reliably separated patients with sepsis and moderate infections: patients with sepsis are characterized by low R relative to the SI level, whereas moderate infections are characterized by the opposite state (Figure 2). (2) R and SI explain the observed differences between patients with sepsis at multiple biological layers. In particular, sepsis pathology, severity, and mortality are associated with a low R, a high SI, and a low R/SI balance (Figures 3, 4, and 6). Finally, (3) the uncoupling between the induction of SI activity and the repression of R activity at the cell-intrinsic level may explain how both hyperinflammation and immunosuppression coexist in the same patient (Figure 2F).

In patients with sepsis, the immune system shows signs of both weak (immune suppression) and exaggerated (excessive inflammation) immune response, each involving a complex cellular reprogramming.^3^ Here, we found that sepsis is characterized by the extreme phenotypes of two distinct cell-intrinsic programs—suppression of R and excessive SI—implying that immune suppression and excessive inflammation could be reprogrammed separately. These are actionable guidelines of blocking inflammation (several drug candidates already being available, such as anti-cytokine antibodies), or amplifying resistance (several candidates such as rIFNg, rIL-7, GM-CSF, etc.), depending on the source of dysfunction in a particular patient (Figure 5). An important aspect needs to be underscored at this point. While the R/SI model can guide the selection of a tailored therapy for each R/SI state, this does not exclude the possibility of using different types of immunotherapeutic approaches depending on the pathophysiological process that has led to a specific R/SI state. For example, it can be envisaged that a state characterized by a strong suppression of resistance without hyperinflammation can be induced through different mechanisms: e.g., a defective IL-12/IFNγ pathway, or overexpression of inhibitory molecules. Such particular immunotypes may respond better to either treatment with recombinant IFNγ or checkpoint inhibitors, to give just one example. Follow-up studies are warranted to define the immunotypes characterizing each of the R and SI dysfunctions, their respective diagnostic markers, and target immunotherapy treatments.

We further demonstrated the advantage of the R/SI framework in patient stratification. In particular, we classified patients with sepsis into immune endotypes based on their R/SI levels and then compared this classification with a former stratification of two extreme endotypes (immunosuppression and MALS).^21^ Using an independent validation cohort (PROVIDE), we showed that the R/SI-based classification presents strong prognostic capacity within each of these former endotypes, highlighting the added prognostic value of the R/SI-based criteria. Furthermore, using the former stratification, many patients with sepsis did not reach the criteria for either immunosuppression or MALS and were therefore described as “unclassified” despite a poor outcome of the disease.^21^ This led to a failure to characterize immunologically a large proportion of patients with sepsis and led to the incapacity to propose appropriate immune-based treatment. Using the R/SI-based classification, we are now able to provide significant prognosis of these previously unclassified patients. We demonstrated that there are plasma protein biomarkers that can identify the R/SI-based endotypes, suggesting translatability of the R/SI-stratification framework into the clinics.

One important aspect to underline is the fact that initially the R and SI programs have been derived from a very distinct context (R – influenza; SI – low-grade chronic inflammation). However, the high co-variation of these programs in sepsis and infection cohorts (Figures S1C–S1E), as well as the link between these programs to clinical measures in different models of sepsis and moderate infections (Figures 2, 3, and 4), strongly argues for their generalizability for understanding sepsis. We note that R and SI are cellular programs—that is, the R and SI states vary at the cell-intrinsic level (Figures 2B, 2F, 3, S3, and S4B)—opening the way to *ex vivo* testing of therapeutic interventions. This is different from recent studies of immune programs that rely on whole-blood transcriptomes^39^^,^^40^^,^^41^^,^^42^^,^^43^ without determining relevance at the cell-intrinsic level. Overall, by leveraging the programs from one context in another context, we improved our understanding of sepsis. This suggests potential reusage of regulatory programs in response to a changing environment, which can be exploited to enhance model generalization in future studies.

Our work opens additional directions for future research. First, the identified sepsis-related pathways, such as BMP2 signaling and elastic fiber formation (Figure S6), would allow the development of therapeutic approaches in sepsis. Second, the transcriptional signatures of R and SI can be used to evaluate immunotherapies of sepsis in an *ex vivo* setting (Figure S5F). Third, future longitudinal studies can provide opportunities to study temporal trajectories of R and SI during sepsis (as in Figures S2D and S2E). For instance, such analysis can show how the trajectory of sepsis deviates from trajectories of moderate infections. Fourth, the R and SI programs can improve the identification of diagnostic markers. For example, the ImmunoSep clinical trial (NCT04990232) relies on mHLA-DR/Ferritin as marker of immunosuppression/hyperinflammation; whereas these markers are indeed associated with R and SI (Figures 3B and S5C), future clinical trials could gain from an unbiased selection of R-immunosuppression and SI-hyperinflammation markers.

Finally, it will be of significant interest to determine how the R and SI states contribute to additional immune-related conditions. For example, R and SI could contribute to trained immunity induction after vaccination or during inflammatory diseases; the R/SI immunotypes during sepsis could be potential risk factors of post-sepsis complications; and the R/SI balance could be used for early prediction of sepsis. Such investigations would require fine-resolution data, not only during infection but also before diagnosis and after recovery, across large cohorts. Overall, the framework of R and SI states can be extended to additional applications in the study of immune-related disease.

### Limitations of the study

Due to the use of observational data in human cohorts, all findings and interpretations in this study should be considered exploratory—that is, it is possible to describe relations and structures in the data, but these patterns cannot be used to make mechanistic interpretations. We adopted this exploratory framework because of the practical limitations of interventions in human cohorts and because of the limitations in mouse sepsis models.^44^ Given the exploratory nature of this study, interpretations should be applied with caution. First, although we identified key types of immune dysregulation, this study cannot identify the driver mechanisms of each dysregulation. Second, although all reported associations have been validated in several complementary tests and independent cohorts, not all covariates were measured—implying that there could be associations due to the effect of extraneous factors on the variables being studied. For instance, this limitation implies that the markers of R and/or SI (identified through associations) could be either driver or passenger mechanisms of R/SI dysregulation; these markers should be therefore used for diagnostics but not yet as therapeutic targets.

Several limitations are related to the currently available datasets. First, the available cohorts mainly include individuals of European ancestry. While we confirmed our findings in an experimental setting (e.g., Figure S4B), it will be important to continue and test the R/SI framework using additional cohorts of populations with different genetic backgrounds. Second, the number of published cohorts that are relevant to our study is limited. For instance, the comparison of sepsis to moderate infections is supported by a relatively small number of cohorts: seven sepsis cohort (datasets #1–#5, #12, and #17) and seven *in vivo* cohorts of moderate infections (datasets #6–#8, #13–#15, and #17). These infections include the leading agents of sepsis (e.g., *S. aureus*, *E. coli*) but also agents that are not typically present in sepsis (e.g., *M. tuberculosis*). Thus, it will be important to continue and test the comparison with additional cohorts, preferably moderate infections with leading agents of sepsis. Third, the comparison of sepsis to moderate infections (Figure 2) primarily relied on differences between cohorts (except from one dataset of patients with UTI^23^). Another caveat is that the moderate-infection cohorts typically include a small percentage of patients with sepsis. In future studies, including both sepsis and non-sepsis infection within each cohort—and a clear patient-level annotation (e.g., sepsis, its severity, and bacterial dissemination into the tissues)—will allow a more comprehensive comparison between sepsis and moderate infections. Fourth, at the current stage, datasets of cell-type-specific profiling across multiple cell types are available only in small cohorts. Such data in larger studies would improve the understanding of the crosstalk between cell types in patients with sepsis. Fifth, the analysis is constrained by the measurements available for each patient. In particular, key features of severe stress and sepsis, which can further enhance our understanding of R and SI, were not measured—e.g., *ex vivo* immune capacity, markers of mitochondrial energy, glycolysis, and the cyclic pentose phosphate pathway as indications of metabolic shifts.^45^^,^^46^ Finally, there is a lack of data for the evaluation of disease tolerance,^47^ because of the experimental difficulties to clearly define the cause of organ dysfunction, tissue damage, and pathogen load. Additional types of data in future cohorts, including an accurate quantification of bacterial load^48^ and tissue damage assessment from plasma cfDNA methylation,^49^ will be valuable to understand disease tolerance in sepsis.

### Conclusions

In the present study, we investigated sepsis pathogenesis in the context of interaction between two transcriptional programs: one aiming to eliminate pathogen invasion (R) and the other associated with SI. Using integrative analysis across multiple sepsis cohorts, we conclude that patients with sepsis are characterized by a molecular fingerprint of a low R program relative to the level of SI. We suggest that the heterogeneity between patients with sepsis, likely explained by the wide diversity of R and SI states, can be used to guide patient stratification to be used in a precision medicine approach toward effective immunotherapy. While the proof regarding the effectiveness of personalized immunotherapy in sepsis still needs to be provided, the ImmunoSep consortium is expected to release soon the results of a randomized trial in which patient stratification based on immune function (hyperinflammation or immunoparalysis) guides the type of immunotherapy administered (NCT04990232). This is likely to represent an important step toward effective immunotherapy in sepsis.

## Resource availability

### Lead contact

Further information and requests for resources should be directed to and will be fulfilled by the lead contact, Mihai G. Netea (mihai.netea@radboudumc.nl).

### Materials availability

This study did not generate unique reagents.

### Data and code availability

• RNA-seq data have been deposited at GEO and are publicly available as of the date of publication. Accession numbers are listed in the key resources table.
• All original code has been deposited at GitHub and is publicly available as of the date of publication. The repository in GitHub is listed in the key resources table.
• Any additional information required to reanalyze the data reported in this paper is available from the lead contact upon request.

## Acknowledgments

We thank all patients in the FUSE and PROVIDE cohorts for their participation. This work was supported by a Competitiveness Operational Program Grant of the Romanian Ministry of European Funds (FUSE) and by European Union Horizon 2020 under grant agreement no. 847422 (ImmunoSep). I.G.-V. is a Faculty Fellow of the Edmond J Safra Center for Bioinformatics at Tel Aviv University. R.B.-L. and G.Y. were supported by the Edmond J Safra Center for Bioinformatics at Tel Aviv University, by the ImmunoSep, and by 10.13039/100010663ERC 637885. M.G.N. was supported by an ERC Advanced Grant (#833247) and a Spinoza Grant of the 10.13039/501100003246Netherlands Organisation for Scientific Research. E.J.G.-B. has received honoraria from Abbott CH, bioMérieux, Brahms GmbH, GSK, InflaRx GmbH, Sobi, and XBiotech Inc.; independent educational grants from Abbott CH, AxisShield, 10.13039/501100022110bioMérieux, 10.13039/501100022344InflaRx GmbH, 10.13039/100004331Johnson & Johnson, 10.13039/100030732MSD, 10.13039/501100012112Sobi, and 10.13039/100017852XBiotech; and funding from the 10.13039/501100007601Horizon 2020 Marie-Curie Project European Sepsis Academy (granted to the 10.13039/501100005187National and Kapodistrian University of Athens) and the Horizon 2020 European Grants ImmunoSep and RISKinCOVID (granted to the 10.13039/501100023710Hellenic Institute for the Study of Sepsis).

## Author contributions

Conceptualization, methodology, and formal analysis, R.B.-L., A.R., G.Y., I.G.-V., and M.G.N.; writing – original draft and review and editing, R.B.-L., A.R., G.Y., I.G.-V., and M.G.N.; funding acquisition, E.J.G.-B., I.G.-V., and M.G.N.; investigation and resources, all authors, and all authors read and approved the final manuscript.

## Declaration of interests

Tel Aviv University has filed a patent application on markers of resistance and systemic inflammation and uses thereof with I.G.-V., R.B.-L., G.Y., M.G.N., and E.J.G.-B. as inventors (PCT/IL2024/050850), which has been filed in the Israel PCT Receiving Office.

## STAR★Methods

### Key resources table

**Table:** 

REAGENT or RESOURCE	SOURCE	IDENTIFIER
**Deposited data**		

Data from humans with sepsis (blood)	This study (the FUSE cohort)	GEO: GSE205672
Data from children with sepsis and SIRS (blood)	Wong et al.^10^	GEO: GSE13904
Data from children with septic shock (blood)	Wong et al.^11^	GEO: GSE26440
Data from children with septic shock (blood)	Wynn, J. L. et al.^9^	GEO: GSE26378
Data from children with S. aureus infection (blood)	Ardura, M. I. et al.^29^	GEO: GSE16129
Data from humans with Tuberculosis infection (blood)	Burel, J. G. et al.^28^	GEO: GSE152532
Data from humans with IAV infection (blood)	Tang, B. M. et al.^27^	GEO: GSE101702
LPS dataset (*in vitro*)	Orozco, L. D. et al.^31^	GEO: GSE38705
scRNA-seq data from humans with SLE and SSc (blood)	Ota, M. et al.^50^	National Bioscience Database Center (NBDC): E-GEAD-397
Time series data from mice with S. aureus and E.coli (blood)	Ahn, S. H. et al.^34^	GEO: GSE33341
Time series data from humans with IAV and Rhinovirus infection (blood)	Zhai, Y. et al.^32^	GEO: GSE68310
Time series data from mice with Ebola infection (liver)	Price, A. et al.^51^	GEO: GSE130629
Time series data from humans with sepsis (blood)	Parnell, G. P. et al.^33^	GEO: GSE54514
Time series data from mice with LPS stimulation (11 tissues)	Takahama, M. et al.^35^	GEO: GSE224146
Septic plasma	Khaenam, P. et al.^52^	GEO: GSE49758
SIRS in neutrophils	Velásquez et *al.*^53^	GEO: GSE123729
*Ex vivo* drug effect	Hu et al.^36^	GEO: GSE144992
scRNA-seq data from humans with sepsis (blood)	Reyes, M. et al.^23^	Broad Institute Single Cell Portal (SCP): SCP548
Data of five bacterial and fungal *ex vivo* infections: *A. fumigatus, C. albicans, P. aeruginosa, S. pneumoniae and M. tuberculosis*.	Le, K. T. T. et al.^30^	GEO: GSE131590

**Software and algorithms**		

Code for calculation of R and SI levels	This study	https://github.com/rachelbl2/Personalized-inflammatory-scores-Pipeline
MSigDB	Broad institute	http://software.broadinstitute.org/gsea/msigdb/collections.jsp
SEEK	Troyanskaya Functional Genomics Laboratory	https://seek.princeton.edu/seek/
Python	Python Software Foundation	https://www.python.org/
NumPy(v1.24.3)	NumPy	http://www.numpy.org/
SciPy(v1.11.4)	SciPy	https://www.scipy.org/
statsmodels(v0.14.1)	statsmodels	https://www.statsmodels.org/
scikit-learn(v1.3.2)	scikit-learn	https://www.scikit-learn.org/
Scanpy(v1.9.6)	scverse project	https://scanpy.readthedocs.io/
Lifelines(v0.27.8)	Cameron Davidson-Pilon	https://lifelines.readthedocs.io/

### Experimental model and study participant details

To investigate sepsis and moderate infections, we conducted analysis of data from several independent cohorts (Table S2; see below for more details). Most cohorts consist of both females and males. Table S2 provides a detailed description of each cohort, including the definition of disease, age and gender characteristics, and exclusion criteria.

#### The FUSE cohort

The FUSE cohort comprises 125 sepsis patients and 284 healthy subjects. Patient characteristics are described in ref.^26^ In brief, included participants enrolled between May 2017 and November 2019 in the Hospital for Infectious Diseases and Pneumology “Victor Babes” Craiova, Romania, and the academic hospital serving Dolj county in south-west Romania; all with declared Romanian ancestry. Inclusion criteria: above 18 years of age, with a diagnosis of sepsis according to the ACCP/SCCM Consensus Conference criteria. Subjects with diagnosis of inherited or acquired immunodeficiency (HIV, chemotherapy or prolonged steroid treatment) were excluded. Healthy controls (>18 years old), with negative medical history and under no prescribed or self-administered medication, were recruited at the Human Genomics Laboratory, University of Medicine and Pharmacy of Craiova. Approval was obtained from the local institutional review boards and all participants signed the informed consent form. Sample collection was performed before initiation of antibiotic therapy. Classification as severe and non-severe sepsis in the FUSE cohort was determined using the quick SOFA (qSOFA) score (GCS <15, respiratory rate >22, systolic BP < 100). Complete SOFA scores using the Sepsis 3 criteria were not available.

All samples were collected within 24h from diagnosis between 07:00-10:00 a.m., and EDTA plasma was separated within 4h since collection at the Human Genomics Laboratory, University of Medicine and Pharmacy of Craiova. Circulatory inflammatory proteins were measured in plasma using the targeted Olink INFLAMMATION panel (v.3021, 92 proteins) with a proximity extension assay (PEA) used by OLINK proteomics.^26^ Protein concentrations were reported as log2 transformation in a normalized protein expression (NPX) scale. Circulating concentrations of ferritin, interleukin IL6, IL1 receptor antagonist (IL1RA), IL18, and IL18 binding protein (IL18BP) were measured with Ella Simple Plex Cartridge Kits (ProteinSimple, San Jose, USA) according to the manufacturer’s protocol.

Gene expression data from PBMCs includes 125 sepsis and 284 controls, and gene expression data for monocytes includes 36 sepsis and 15 controls. RNA extraction and RNA-sequencing of PBMCs and monocytes were performed as follows. The PBMC fraction obtained by density centrifugation of blood diluted 1:1 in phosphate-buffered saline (PBS)-buffer over Ficoll-Paque (GE Healthcare). Cells were washed three times in cold PBS and resuspended in RPMI 1640 (Dutch modified) supplemented with 50 mg/L gentamicin, 2 mM L-glutamin (GlutaMAX), and 1 mM sodium pyruvate (Thermo Fisher Scientific). Pan Monocyte Isolation Kit (Miltenyi Biotec 130-096-537) was used to isolate monocytes from PBMCs by depletion of non-monocytes (negative selection). RNA was isolated using the RNeasy mini kit (QIAGEN), and only RIN>7 samples were used for subsequent RNA-Seq. The RNA-Sequencing library was prepared by using the MGIEasy RNA Library Prep kit (MGI Tech), and strand-specific RNA sequencing was performed using the DNBseq platform (paired-end, read length 100 bp), averagely generating 24.05M (std ±0.54M) reads per sample.

##### RNA-seq preprocessing

We removed the reads mapped to rRNA to get raw data. We used the SOAPnuke v1.5.2 software (parameters: -l 15 -q 0.5 -n 0.1) to filter reads, specifically removing reads with adaptors, reads in which unknown bases are more than 10%, and reads with low quality (%bases of quality lower than 15 is greater than 50%). As evidence for the quality of sequencing, we observed that total clean reads were on average 23.99M (±0.54M) reads per sample; the averaged clean reads ratio was 99.7%; the average of Q20 was 98.3% (±0.29%) and the average Q30 was 91.9% (±0.99%). After reads filtering, we mapped clean reads to reference genome using the Bowtie2 v2.2.5 software. We observed high quality of mapping: the average mapping ratio with reference genome was 93.63%, the average mapping ratio with gene was 78.71%, and a total of 19,387 genes were detected. The uniformity of the mapping results for each sample suggested that the samples are comparable. Gene expression levels (FPKM) were calculated with RSEM v 1.2.12.

#### Datasets included in this study

We used several gene-expression datasets for the analysis of sepsis and moderate infections. For the FUSE, the preprocessing is detailed above. For all remaining datasets, we downloaded the preprocessed data. For all datasets, additional preprocessing steps were the handling of missing data, log transformation and standardization, as detailed in STAR Methods. Table S2 provides a detailed characterization of each of the datasets.

##### Comparison of sepsis to moderate infection in Figures 2A–2D

We used the following datasets for the comparison of sepsis to moderate infections in Figures 2A–2D: 1) datasets of sepsis patients, in which the percentage of diagnosed sepsis is 100% (datasets #1-#5). 2) datasets of infection without sepsis (referred to as ‘moderate infection’), in which we require that the expected percentage of sepsis would be low (<5%; datasets #6-#10).

###### Datasets of sepsis

(1) *Sepsis, PBMCs* (the FUSE cohort^26^): data from 284 controls and 125 sepsis individuals, as described above. Data was deposited in GEO accession GSE205672 (secure token: ojkpcoochlgxhez).
(2) *Sepsis, monocytes* (the FUSE cohort^26^): data from 36 sepsis and 15 controls, as described above. Data was deposited in GEO accession GSE205672 (secure token: ojkpcoochlgxhez).
(3) *Sepsis in blood dataset*: Whole blood expression data from 18 healthy and 52 sepsis samples (GEO accession GSE13904).^10^
(4) *Septic shock in blood*dataset I (referred to as SS-I): Whole blood expression data from 98 children with septic shock and 32 healthy controls (GSE26440).^11^
(5) *Septic shock in blood*dataset II (referred to as SS-II): Whole blood expression data from 82 children with septic shock and 21 healthy controls (GSE26378).^9^

###### Datasets of moderate infection

(1) *Influenza A virus (IAV) dataset*: Whole blood expression data from 63 adults with moderate IAV infection and 52 healthy controls (GSE101702).^27^
(2) *M. Tuberculosis* (TB) *dataset*: Blood transcriptome in a cohort of 26 active TB samples and 11 healthy samples that were collected at diagnosis (before the start of treatment) and post-treatment (GSE152532).^28^
(3) *S. aureus (moderate) infection dataset*: PBMCs transcriptomes of 10 healthy controls and 8 *S aureus* patients with invasive *in vivo* infection but no bacteremia (GSE16129).^29^ Individuals with bacteremia were excluded from this cohort.
(4) *LPS dataset*: Expression in primary macrophages of mouse inbred strains. 89 samples from different strains were exposed to bacterial lipopolysaccharide (LPS) and 86 samples were mock-treated and use as controls (GSE38705).^31^
(5) A dataset of five bacterial and fungal *ex vivo* infections: Transcriptomic responses of human PBMCs from eight individuals with 5 pathogenic stimulations at 4h post infection (32 samples). Stimulations are: *A. fumigatus, C. albicans, P. aeruginosa, S. pneumoniae and M. tuberculosis.* Stimulation with RPMI was used as a control (8 samples) (GSE131590).^30^

####### Selection of datasets

Datasets were selected in an unbiased manner from the GEO repository, as follows: (i) For sepsis, we included all human datasets with at least 30 sepsis individuals and 15 controls. (ii) For moderate infections, we included all human datasets with at least 25 infected individuals and 10 controls. In case of more than one dataset for the same pathogen (e.g., for influenza virus infection there were many optional datasets), we chose the largest dataset in which clinical information was available. We specifically searched for the bacterial, viral, fungal pathogens that are common in sepsis. We specifically searched for cohorts (or sub-cohorts) in which the expected percentage of sepsis is low. (iii) In both sepsis and moderate infection datasets, we selected only datasets of mRNA profiling, and filtered out datasets with comorbidities (e.g., super-infections, genetic mutations, and specific characterization of an additional disease) or known interventions (e.g., vaccines) (detailed in Table S2). We further included datasets #8 and #10 due to their high relevance. The expected percentage of sepsis within each dataset is reported in Table S2.

##### Comparison of sepsis to moderate infection (and other conditions) in Figures 2E, 2F, and S2

(1) *Autoimmune disease dataset* (SLE and SSc): Isolated cells from 26 CD45^+^ cell types derived from blood samples across 63 healthy subjects, 60 SLE patients and 45 SSc patients (E-GEAD-397).^50^
(2) *Sepsis: Time series data of human sepsis.* Expression profiling of whole blood for up to 5 days for 35 sepsis patients and 18 healthy controls (GSE54514).^33^
(3) *In vivo* infection without sepsis: Time series data of murine S. aureus and E. coli *in vivo* bacterial infections: Expression profiling of blood for up to 12 h (S. aureus) or 24 h (E. coli). For S. aureus, 78 infected samples and 29 control samples were included. For E. coli, 40 infected samples and 10 controls samples were included (GSE33341).^34^
(4) *In vivo infection without sepsis: Time series data of IAV and Rhinovirus in vivo human infections*: Blood expression data from 70 healthy adults that developed respiratory infection (45 IAV infection and 25 rhinovirus infection) before the infection (controls) and at 4 timepoints over 6 days after the initiation of the infection’s symptoms (GSE68310).^32^
(5) *In vivo infection without sepsis: Time series data of murine in vivo Ebola infection*: Transcriptional profiles from liver from 10 Collaborative Cross mice infected with mouse-adapted Ebola virus (MA-EBOV) in 3 time points; a total of 30 infected samples (GSE130629).^51^
(6) *Sepsis: Time series data of a murine model of sepsis* (*in vivo* LPS stimulation): Transcriptional profiles from 11 tissues of mice stimulated with LPS in 7 time points, 4 mice for each time point; a total of 308 samples (GSE224146).^35^
(7) *Dataset of sepsis and infection without sepsis, scRNA-seq*^23^: The data includes scRNA-Seq of 19 healthy controls, 10 urinary tract infection (UTI) patients showing clear symptoms of sepsis (i.e., persistent organ dysfunction), and 10 UTI patients that are classified as moderate infection (leukocytosis but no organ dysfunction). The two groups are referred to as URO and Leuk-UTI in the original data publication; the URO consists primarily of *E. coli* (60%) and *Enterobacter cloacae* complex (20%) infections, and the Leuk-UTI consists primarily of *E. coli* (50%) and *K. pneumoniae* (20%) infections. All disease patients were enrolled within 12 h of presentation to the emergency department and within 12 h of antibiotic treatment, and most recruited subjects are older adults.^23^ We analyzed subpopulations of monocytes, T, B and NK cells. Monocytes: 36,206 monocytes that were previously categorized into four subpopulations (MS1-MS4). B cells: 6603 cells in two subpopulations BS1, BS2. T cells: 25472 cells in two subpopulations TS1,TS2. NK cells: 6101 cells in two subpopulations NS1,NS2. Dendritic cells were not analyzed due to their low numbers of cells in sepsis. Each subpopulation was analyzed separately, as demonstrated in Figures 2F–I, II, S3A, and S3B for the case of monocytes.

In this scRNA-seq dataset, the responses of R and SI were defined for each cell subpopulation of each individual subject. In particular, for each individual i and each cell subpopulation j, the molecular ‘*R response’* (or ‘*SI response’*) was calculated by comparison of R levels (or SI levels) of all cells in subpopulation j from patient i against the R levels (or SI levels) of all cells in subpopulation j from all control subjects. The R (or SI) response is defined as log_10_ t test *p* value, signed according to the direction of response (positive/negative for increase/decrease in the averaged R (or SI) levels).

To select the time series datasets #12-#16, we used the same criteria ii and iii above, but searched for both mouse and human datasets. The expected percentage of sepsis within each of the additional datasets is reported in Table S2.

##### Additional datasets

(1) *Sepsis (PROVIDE).* PROVIDE consists of 223 sepsis patients. PROVIDE is a randomized controlled immunotherapy trial carried out in patients diagnosed with sepsis according to the Sepsis-3 criteria,^1^ caused by either community-acquired pneumonia, healthcare-associated pneumonia, ventilator-associated pneumonia, acute cholangitis, or primary bloodstream infection. Patients were recruited in 14 study sites in Greece, in accordance with the applicable rules concerning the review of research ethics committees and informed consent (EudraCT 2017-002171-26; approval by the National Ethics Committee of Greece 78/17; approval IS 75-17 by the National Organization for Medicines of Greece; Clinicaltrials.gov registration NCT03332225). In order to classify patients into immunological endotypes, serum ferritin concentration and mHLA-DR expression were measured. Patients with serum ferritin concentrations of 4420 ng/mL or above were classified as ‘hyperinflammation’ (MALS),^4^ regardless of their mHLA-DR expression. Patients with mHLA-DR expression of 5000 antibodies bound per cell (Ab/cell) or less and ferritin concentrations lower than 4420 ng/mL were classified as having ‘immunosuppression’.^21^^,^^37^^,^^38^^,^^54^ If neither the criteria for MALS nor immunosuppression were fulfilled, patients were categorized as ‘unclassified’.
(2) *Septic plasma*: Stimulation of granulocytes, dendritic cells and PBMCs from healthy subjects with plasma from either sepsis patients or healthy subjects. Included are 76 profiles of plasma stimulations, among them 27 profiles of stimulation with plasma of healthy subjects and 49 profiles of stimulations with plasma from sepsis patients (GSE49758).^52^
(3) *S. aureus (all)*: PBMCs transcriptomes of 10 healthy controls and 46 *S aureus* patients with invasive infection (GSE16129).^29^ Patients in this cohort likely include both moderate and severe infection, local and disseminated infection, sepsis and non-sepsis infection (no annotation of specific individuals).
(4) *SIRS, neutrophils*: Neutrophils isolated from blood of 11 healthy adults and 16 SIRS patients. All SIRS patients were confirmed to have negative blood culture. SIRS was compared to 15 sepsis patients in this dataset (GSE123729).^53^
(5) *SIRS, blood*: Whole blood expression data from 18 healthy individuals, 27 SIRS patients, and 52 sepsis patients. All SIRS patients were confirmed to have negative blood culture (GSE13904).^10^

### Method details

#### Selection of the R and SI programs

To study transcriptional immune programs in a systematic manner, we collected a set of 76 programs, including: 1) All programs from the Immune Knowledge Base collection^55^ that include the term ‘response’ in their name (20 programs). 2) All genesets from the ImmuneSigDB collection (the C7 subcollection of MSigDB, https://www.gsea-msigdb.org/gsea/msigdb/) that were originally generated based on data in PBMCs, excluding sepsis datasets (50 programs). 3) Additional programs that were originally developed or validated based on associations with clinical data, including R and T,^24^ IM1 and SI,^25^ and SAS-I/MAS-1^42^ (6 programs). Each program was evaluated using three criteria, as follows.

##### Critarion 1

*The response to pathogens.* We evaluated *in vivo* infections compared to sterile inflammation using datasets #6, #7, #20, #21 and #22 (Table S2). For each dataset and each program, we compared disease against healthy in two steps: 1) for each individual, we calculated the level of the program (geneset programs: by averaging the gene set; gene-weights programs [R,T, IM1, SI]: as detailed below); 2) a Wilcoxon signed rank test statistics is used to compare the program level between disease and healthy subjects (positive/negative for upregulation/downregulation in disease compared to healthy; scores are presented in Figure S1A).

##### Critarion 2

*The ‘covariation in sepsis’ score.* We aimed to evaluate the relevance of a program to sepsis, specifically by testing how well the genes of a given program are co-varying (co-expressed) in sepsis. The analysis was performed using the SEEK algorithm,^56^ which integrates transcriptomes from multiple datasets and analyzes them in a systematic manner. The SEEK’s input is a query geneset, and the output is a ranking of all datasets according to the covariation of the query set in each transcriptome dataset. We used each of the 76 program separately as a query geneset (for a continuous gene weighting, the 100 top-weight genes were used as the geneset) and ranked all blood non-cancer datasets (437 datasets). Thus, for each of the 78 programs, the output is a covariation-based ranking of each of the 437 datasets. Among the 437 datasets under study, seven datasets are sepsis datasets. For the task of detecting sepsis datasets based on their covariation ranking, we defined the ‘actual positives’ as the seven sepsis datasets, and the ‘actual negatives’ as the remaining 69 datasets. For a given program, the ‘predicted positives’ are the datasets with a ranking ≤c, the ‘predicted negatives’ are the datasets with a ranking >c, the ‘true positives’ are the actual positives that are also predicted positives, *precision* for a certain c is the number of true positives divided by the number of predicted positives, and *recall* for a certain c is the number of true positives divided by the number of actual positives. F1 is a combination of precision and recall: for a certain cutoff *c* and a given program, a higher F1 score implies a better accuracy of predicting the sepsis datasets based on the covariation of the program. For each program, we calculate the F1 scores when using the ranking of each sepsis dataset as the c cutoff. The ‘covariation in sepsis’ score of a program is the average of the F1 scores across the seven cutoffs (reported in Figure S1B).

##### Critarion 3

*The ‘covariation in infections’ score.* This score is the same as the ‘covariation in sepsis’ score with one difference: the ‘actual positives’ group consists of all 45 infection datasets (rather than only 7 sepsis datasets). Thus, the F1 scores was calculated when using the ranking of each infection dataset as the c cutoff, and the ‘covariation in infections’ score is the averaged F1 scores across all 45 cutoffs. A higher covariation in infections score implies a better accuracy of predicting the infections datasets based on the covariation of a program (reported in Figure S1B).

#### Calculation of R level, SI level, and the R/SI balance

For a given dataset, we first preprocessed the data and then calculated SI levels, R levels, and the R/SI-balance score.

##### Data preprocessing

Preprocessing is applied in four steps. 1) Gene filtration and imputation. For datasets with a low number of missing values (<1%), genes with at least one missing value were removed. For datasets with medium number of missing values (>1%, <15%), genes with high amount of missing data (>75%) were filtered out and then KNN imputation (K = 5) was applied. For datasets with high number of missing values (>15%), we removed genes with high number of missing values (>75%) with no subsequent imputation. All zero counts were treated as missing values. 2) Log2-transformation. 3) Sample-level standardization: each sample is centered and divided by standard deviation across genes. 4) Gene-level standardization: each gene is centered and divided by standard deviation based on the distribution in the healthy samples – that is, only the healthy samples are used for the calculation of the standardization that is subsequently applied on all samples. For individual i, the output of this preprocessing is a gene expression vector $Z_{i}$.

##### Calculation of SI levels

As previously described,^25^ the calculation of SI (originally referred to as IM2 in ref.^25^) must take into consideration the confounding effect of an IM1 factor. In a model for a given individual (or sample) i, its expression profile $Z_{i}$ is expressed as a weighted sum of the effects of each gene with respect to IM1 and SI: $Z_{i}={b_{i}+s}_{IM1}^{i}V_{IM1}+s_{SI}^{i}V_{SI}$ (Eq. 1). For a given individual *i*, $Z_{i}$ is the input vector of pre-processed gene-expression levels of individual i; the vectors $V_{IM1}$ and $V_{SI}$ are the pre-defined “gene weights” for IM1 and SI, respectively, which were originally defined in ref.^25^; $s_{SI}^{i}$ and $s_{IM1}^{i}$ are the output: $s_{SI}^{i}$ is the inferred “SI level” of individual i, and $s_{IM1}^{i}$ is the inferred “IM1 level” of individual i. $b_{i}$ is a constant. The original analysis of IM1 and SI in ref.^25^ relied on healthy obese individuals, which allow to define systemic inflammation in the absence of pathogen (150 healthy, obese individuals from the 300-OB cohort^25^). In accordance, the gene weights of IM1 and SI ($V_{IM1}$ and $V_{SI}$) reflect a general inter-individual variation in immunometabolism. Results showing that the inferred SI level is indeed associated with systemic inflammation are detailed in ref.^25^. We interchangeably use the terms ‘SI level’ and ‘SI state’.

##### Calculation of R levels

As previously described,^24^ the calculation of R must take into consideration the confounding effect of an additional factor, referred to as T. In a model for a given individual (or sample) i, its expression profile $Z_{i}$ is expressed as a weighted sum of the effects of each gene with respect to T and R: $Z_{i}={b_{i}+s}_{T}^{i}V_{T}+s_{R}^{i}V_{R}$ (Eq. 2). For a given individual *i*, $Z_{i}$ is the input vector of pre-processed gene-expression levels of individual i; the vectors $V_{T}$ and $V_{R}$ are the pre-defined “gene weights” for T and R, respectively, which were originally defined in ref.^24^; $s_{T}^{i}$ and $s_{R}^{i}$ are the output: $s_{T}^{i}$ is the inferred “T level” of individual i, and $s_{R}^{i}$ is the inferred “R level” of individual i. $b_{i}$ is a constant. Results showing that the inferred R level is indeed associated with resistance are detailed in Cohn et al., 2022.^24^ We interchangeably use the terms ‘R level’ and ‘R state’.

For the calculation of R and SI levels, Z and V are n-length vectors where n is the number of genes. The upper bound of n is the number of genes for which the predefined gene weights are available, but for each specific sample the number of genes could be lower, depending on the number of genes that were actually measured. Conversion between mouse and human genes was performed with the org.Mm.e.g.,.db Bioconductor package using the list of one-to-one homologs from the complete MGI list of human-mouse homologs.

##### Calculation of the R/SI-balance score

As R and SI levels could be in different scales, we first applied standardization of R and SI levels. Standardization was performed based on the healthy controls – i.e., subtracting the mean level (centering) and dividing by the standard deviation of the control subjects (applied separately for R and SI). The ‘R/SI balance score' was calculated by subtracting the (standardized) SI level from the (standardized) R level (Figure 2D).

#### Validity of the R and SI programs

We aimed to evaluate to what extent the R and SI programs are relevant to human blood samples. By using the predefined gene weights ($V_{R}$, $V_{SI}$) in the analysis of independent cohorts, we could assess the relevance of a program in various conditions. The analysis was applied on data from the human circulation of healthy subjects as well as *in vivo* viral and bacterial infections ranging from moderate to high severity (datasets #1,#2,#6, #20, Table S2). Two evaluation approaches were applied:

##### Approach 1

*Analysis of inter-individual variation*. We evaluated how well program levels predict inter-individual variation in gene expression. The analysis was applied separately in each cohort. For each gene g, we applied the following analysis: in step 1, we calculated the SI and R levels of each individual in the cohort, holding out the data of gene g, using $Z_{i}={b_{i}+s}_{X}^{i}V_{X}+s_{Y}^{i}V_{Y}+\varepsilon_{i}$ (Eq. 3). $Z_{i}$ is the measured gene expression profile of individual i excluding gene *g*, $V_{X}$ and $V_{Y}$ are the predefined gene weights excluding gene g, and ($s_{X}^{i},s_{Y}^{i})$ are the output program levels of individual i. X and Y are either R and T, or alternatively, SI and IM1. In step 2, we evaluated the quality of prediction of the expression of gene g from the R and SI levels (a *joint model*): $Z_{g}={\beta_{c}^{g}+\beta }_{R}^{g}S_{R}+\beta_{SI}^{g}S_{SI}+\varepsilon_{g}$ (Eq. 4), where $Z_{g}$ is the gene expression profile of gene g across all individuals, $S_{R}$ and $S_{SI}$ are the inferred levels of programs R and SI (respectively) from step 1 across individuals, and $\varepsilon_{g}$ is the noise for gene g. In addition, we use an *R-only model:* $Z_{g}={\beta_{c}^{g}+\beta }_{R}^{g}S_{R}+\varepsilon_{g}$ (Eq. 5), and an *SI-only model:* $Z_{g}=\beta_{c}^{g}+\beta_{SI}^{g}S_{SI}+\varepsilon_{g}$ (Eq. 6). Overall, we evaluated three models (joint, SI-only, R-only). We repeated the analysis 10 times; in each repeat, 10% of the genes (without replacements) are held out for the calculation of R and SI levels in step 1 (Eq. 3), and the regressions in step 2 (Eqs. 4–6) are calculated only for the held-out genes. For comparison, the entire analysis was applied on permuted data, which was performed by reshuffling the expression and program levels for the calculations in Eqs. 4–6.

Based on this framework, two scores were calculated: (i) For each gene, each model and in each dataset, the *p*-value of the regression (Eqs. 4,5, or 6). As exemplified in Figure S1C-I, the comparison of *p*-values from real and permuted data allows to identify the genes with empirical *p* < 0.05. For a given dataset and a given model, the ‘*percentage of significant genes*’ is the percentage of genes with empirical *p* < 0.05 (reported in Figure S1C). (ii) For each gene, each model and in each dataset, the $R^{2}$ of the regression (Eqs. 4,5, or 6). This $R^{2}$ is referred to as the ‘*percentage of explained individual variation’*. For the task of detecting real genes based on the percentage of explained individual variation ($R^{2}$), the fractions of genes with $R^{2}>c$ in real and permuted data are the true positive rate (TPR, lower bound) and false positive rate (FPR), respectively. For example, using the joint model in PBMCs of sepsis patients, we observe that 0.91 of the genes obtained $R^{2}>0.05$ using real data (TPR = 0.91) and only 0.04 of the genes obtained $R^{2}>0.05$ using permuted data (FPR = 0.04) (Figure S1D-I). Precision, recall and F1 values are calculated based on these TPR and FPR values (reported in Figure S1D).

##### Approach 2

*Analysis of inter-gene variation.* We evaluated how well the gene weights of a program predict the variation between genes within each individual. The analysis was performed separately for the gene weights in the R/T programs and the SI/IM1 programs. Using each individual separately, we evaluated the regression between the predefined gene weights (independent variables) and the observed gene expression levels (dependent variable). For a given individual*,* we used the $R^{2}$ of this regression as the evaluation metric, which reflects the percentage of explained inter-gene variation (within individual). Permuted data analysis was performed by permutation of the predefined gene weights before the calculation of the regression. For each dataset separately, we calculated the percentage of individuals with empirical *p* < 0.05 (Figure S1E).

#### Functional evaluation of programs R and SI

##### Functions of R and SI

We aimed to evaluate the relevance of R and SI to bacterial infections. To that end, we defined sets of SI and R markers. The SI markers are the top 250 genes that co-vary with the SI level in bacterial infections, and the R markers as the top 250 genes that co-vary with the R level in bacterial infections (co-variation is the ‘percentage of explained individual variation’ score, averaged across datasets #1,#2,#20). Table S1 reports hyper-geometric enrichments of these markers.

##### Co-expression of R and SI

The analysis was performed using the SEEK algorithm,^56^ which integrates transcriptomes from 5210 datasets and analyzes them in a systematic manner. The SEEK’s input is a query geneset (here, 100 top-weight R genes and 100 top-weight SI genes). The analysis was applied separately on the query geneset of R and SI. The SEEK’s output is 1) the co-expression of the query set in each dataset (the ‘dataset’s co-expression score’), and 2) the co-expression of each gene with the query geneset across the entire collection of datasets (the ‘gene’s co-expression score’). Enrichment of 100 top co-expressed datasets was performed using the MeSH terms and enrichment of 100 top co-expressed genes was performed using the GO annotation (reported in Table S1).

#### Analysis of the PROVIDE cohort

##### Calculation of R and SI levels in PROVIDE

The R and SI levels were originally calculated using gene expression data based on a 2D map of gene weights.^24^^,^^25^ Using the FUSE cohort, we calculated the weights of 92 protein (rather than genes) in a 2D protein map where R and SI as the main axes – particularly, we used the correlations with R and SI levels across the FUSE individuals as the weights of proteins in the R-SI protein map. As independent confirmation for the quality of this FUSE protein map, we calculated the correlation between each clinical parameter and each protein in the PROVIDE cohort. In Figures 4B and 4C, the FUSE protein map is presented as a scatterplot where each protein is a dot in a 2D space. The color coding of this map is according to the correlations of each protein with a certain clinical parameter in the PROVIDE cohort. This visualization highlights a clear organization of the map in various directions, confirming the validity of the FUSE protein map in the independent PROVIDE cohort. Encouraged by this observation, the FUSE protein map was used as the basis for the calculation of R and SI levels of each PROVIDE patient. In the same way that we used transcriptome data relying on the map of gene weights for the calculation of R and SI levels, in the PROVIDE cohort we used the map of protein weights for the calculation of R and SI levels.

##### Survival analysis for the R/SI-based endotypes

Kaplan–Meier plots were constructed to evaluate differences in survival rate among the R/SI-based endotypes. Survival was measured from the date of hospital admission to date of discharge (restricted to 28 days). Log rank test was performed to evaluate the significance of survival differences between endotypes. The COX proportional hazards model estimated the hazard of death (with age and gender as additional covariates), either for all sepsis patients or for specific subtypes of sepsis identified using the ferritin (MALS) and mHLA-DR (immunosuppression) biomarkers.

##### Biomarkers of R, SI and the R/SI balance

As a marker for R, we used the average of CXCL11 and IFNγ plasma proteins. As a marker for SI, we used the average of IL6 and IL8 plasma proteins. Calculation of the R/SI balance was done as described above but using the protein markers of R and SI rather than the transcriptome-based calculation. The biomarkers were selected as follows: Using the FUSE cohort, the plasma proteins IFNγ and CXCL11 were suggested as top markers of R levels, and the plasma protein IL6 and IL8 were suggested as top markers of SI levels (Figure 3B). These markers were also confirmed in the PROVIDE cohort (Figure S5A).

#### Functional properties of the R/SI balance

We aimed to identify key functional properties of sepsis that set it apart from moderate infections (Figure S6; Table S5). The analysis was applied in several steps. First, we calculated the correlation of every gene to the R/SI-balance score across all individuals (applied separately in each sepsis dataset). Next, for each gene, the mean correlation was calculated across all cohorts. This average is the general ranking of genes as markers of the R/SI balance score (Table S5). The ‘top ranked genes’ are those with average correlation >0.6 for good balance and <-0.45 for impaired balance. Third, among the top ranked genes, we selected the 300 ‘top markers’ according to their correlations in monocytes (i.e., 300 top markers of a good R/SI balance and 300 markers of an impaired R/SI balance; Table S5). Finally, for a given functional class and a given set of R/SI balance markers, the enrichment *p*-values were calculated using a hyper geometric test. We tested all functional classes in the Reactome and MSigDB’s CGP collections. All reported results are FDR-adjusted *p*-values (q values, Figure S6B). To follow up the prediction of quiescence, we used a quiescence gene set from Cuitiño et al. 2019^57^; these genes were found as markers in quiescent (G0) embryonic cells, as opposed to cycling (G1, G1-S, and S-G2-M) embryonic cells (embryonic days 10.5 (E10.5), E11.5, and E13.5).

## Supplemental information

Document S1. Figures S1–S6

Table S1. The R and SI programs and their functional characterization, related to Figure 1 Column 1 indicates the type of enrichment (genesets or GEO) and column2 indicates the enriched program (R or SI). Enrichments of R and SI in genesets: Presented are minus log 10 q-values of all significantly enriched genesets, for the enrichment of R markers or SI markers (hyper-geometric test, FDR corrected). The selection of SI and R markers is detailed in Methods. Co-expression of R and SI in the GEO compendium: As detailed in Methods, the analysis was performed using the SEEK algorithm, which integrated transcriptomes from 5210 datasets in GEO in a systematic manner. The table reports all enriched functional categories, together with their hyper-geometric FDR-corrected *q*-value, for the R program and SI program. Column 1 indicates the enrichment type (top co-expressed datasets or genes) and column 3 indicates which functional categories was used.

Table S2. Datasets included in this study, related to Figures 1, 2, 3, 4, and 6

Table S3. Relations of R, SI, and the R/SI-balance with plasma proteins, related to Figure 3 Reported are the Pearson's correlations of each protein (column 1) with the R/SI balance, SI and R in PBMCs (columns 3-5) and monocytes (columns 6-8), as well as the percentage of variation in plasma proteins that is explained by the R/SI balance (PBMCs: column 9-10, monocytes: columns 11-12). Column 2 indicates top markers of each program. All correlations are reported for the FUSE cohort, where the SI and R levels were calculated using gene expression in either PBMCs or monocytes.

Table S4. CPH models, related to Figures 4 and 6 Analysis of 28-days mortality in the PROVIDE cohort with a Cox proportional hazards [CPH] model. Top: CPH model for the R/SI balance score as a quantitative variable. Presented are hazard ratio (HR) and 95%-confidence interval (columns 4-6) for the prediction of 28-days mortality using the R/SI balance as a continuous variable (column 1) with additional parameters of age and gender (column 2). The model was independently fit to each group of patients: either all sepsis patients in PROVIDE (“All individuals”) or each previously defined group (MALS, immunosuppression and unclassified, column 3). P-values for the R/SI balance score are indicated in column 7. Middle and bottom: CPH model for the R/SI-based endotypes. Reported are HR and 95%-confidence interval (columns 4-6) for each endotype (column 1) as predicted by a Cox proportional hazards model with age and gender covariates (column 2). The model was independently fit to each endotype. For the SI-high endotype, the variable equals 0 for moderate R/SI balance and 1 for SI-high. For the severe R/SI balance endotype, the variable equals 0 for moderate R/SI balance and 1 for severe R/SI balance. The model was independently fit to each group of patients: either all sepsis patients in PROVIDE (“All individuals”) or for specific previously defined groups (MALS, immunosuppression and unclassified, column 3). P-values for the endotype are indicated in column 7. HR values for the age and gender factors are not indicated. The R and SI were approximated in two different ways: using global expression as in Figure 6A (middle) or using protein biomarkers: R – the average of IFNγ and CXCL11; SI – the average of IL6 and IL8 (bottom).

Table S5. mRNA markers for the R/SI balance, related to Figure 4 Reported are the correlations of each gene with the R/SI-balance (columns 2-7), resistance (columns 8-13) and systemic inflammation (columns 14-19). For each program (R and SI), reported are the correlations in different cohorts (indicated in titles 1 and 2; see details in Table S2). Column 2 provides the averaged correlation with the R/SI-balance score across all cohorts; genes with high absolute correlation are denoted ‘top ranked genes’. Genes were ordered by their R/SI-balance score in monocytes (column 3), highlighting the HLA-DR genes among the top R/SI biomarkers in monocytes. Among the top-ranked genes, the rightmost column (column 20) indicates the top genes of highest positive correlations in monocytes (the selected markers of a good R/SI balance) and the top genes of strongest negative correlations in monocytes (the selected markers of an impaired R/SI balance). Thus, the R/SI-balance markers were selected based on their scores across all cohorts (column 1) and specifically their scores in monocytes (column 3); these markers were used as two gene sets in the subsequent functional analysis (Figure S6B).

Table S6. Patient characteristics of the PROVIDE cohort according to the SI/R-based endotypes, related to Figure 6 Data are presented either as n (%) or by mean (SD). All measures and scores are on day 1 of sepsis diagnosis. Statistical analysis is t test unless stated otherwise: ∗χ^2^ test. †Log-rank test.

Document S2. Article plus supplemental information
