# Supplementary material for: Sepsis pathogenesis and outcome are shaped by the balance between the transcriptional states of systemic inflammation and antimicrobial response
Source: Cell Rep Med. 2024 Nov 19;5(11):101829. doi: 10.1016/j.xcrm.2024.101829 (PMC11604535; doi:10.1016/j.xcrm.2024.101829)

Cell Reports Medicine, Volume *5*

Supplemental information

Sepsis pathogenesis and outcome are shaped by the balance between the transcriptional states of systemic inflammation and antimicrobial response

Rachel Brandes-Leibovitz, Anca Riza, Gal Yankovitz, Andrei Pirvu, Stefania Dorobantu, Adina Dragos, Ioana Streata, Isis Ricaño-Ponce, Aline de Nooijer, Florentina Dumitrescu, Nikolaos Antonakos, Eleni Antoniadou, George Dimopoulos, Ioannis Koutsodimitropoulos, Theano Kontopoulou, Dimitra Markopoulou, Eleni Aimoniotou, Apostolos Komnos, George N. Dalekos, Mihai Ioana, Evangelos J. Giamarellos-Bourboulis, Irit Gat-Viks, and Mihai G. Netea

Supp. Figure 1. Characterization of the R and SI programs, related to Figure 1.

(A,B) Selection of immune programs.

(**A**) Response of candidate programs to infections and SIRS. The analysis was performed in datasets #6, #7, #20, #21, #22, as detailed in **Table S2**. The effect of disease on the program level is color coded for each program (column) and each condition (row) (red/blue indicates upregulation/downregulation, Wilcoxon signed rank test statistic). The matrix shows that among all candidate programs, only the 'resistance' (R) program is upregulated in response to all three types of infections but is not upregulated in the two SIRS datasets. Thus, only R presents a response to pathogens in a broad and specific manner. Panel G provides a detailed visualization of the two SIRS datasets for the R and SI programs.

(**B**) Co-expression of each candidate program during infections in general and sepsis in particular. The analysis was performed across 437 datasets from GEO that were retrieved using the SEEK algorithm, as detailed in **Methods**. For each candidate program (a dot), the scatter plot shows the 'covariation in sepsis' score (x axis) and the 'covariation in infections' score (y axis). Selected programs are indicated. For the covariation in sepsis, the 'systemic inflammation' (SI) program outperformed all other programs in its 'covariation in sepsis' score. It was therefore selected for the analysis in this study. For the covariation in infections, several programs obtained high covariation-in-infections scores at similar levels, such as the resistance (R) program and the response to yellow-fever vaccine; among these top-performing programs (and also in general), only the 'resistance' program presents a specific response to pathogens (see in A). We therefore used the R program for the analysis in this study.

(**C-E**) **Validity of the R and SI programs**. The analysis was applied on six cohorts: Healthy, PBMCs (284 healthy, dataset #1), healthy, blood (52 healthy, dataset #6), S aureus (46 infected, dataset #20), IAV, blood (63 infected, dataset #6), sepsis, PBMCs in the FUSE cohort (125 infected, dataset #1), sepsis, monocytes in the FUSE cohort (36 infected, dataset #2) (see **Table S2** for details). All metrics are detailed in **Methods**.

(**C**) Prediction of gene expression in held-out data (the p value score). (**C-I**) An example. Box plots for the minus log p-value metric across all genes. The plots present the analysis of real data and permuted data (x axis), for the joint model, using the FUSE cohort of PBMCs from sepsis patients. The red line indicates empirical p = 0.05. The percentage of significant genes in which the model presents empirical p <0.05 is specified (red line). (**C-II**) Percentage of significant genes in several cohort. The table reports the percentage of significant genes with empirical p<0.05 using each dataset (rows) and each model (columns: SI-only model, R-only model, and a joint model). The calculation of the percentage of significant genes is exemplified in panel C-I for one specific cohort.

(**D**) Prediction of gene expression in held-out data (the $\boldsymbol{R^2}$ score). (**D-I**) Box plots for the R^2 metric across all genes. The plot presents the analysis of real data and permuted data (x axis), for the joint model, using the FUSE cohort of PBMCs from sepsis patients. Cutoff of $R^2 = 0.05$ is indicated as red line. Genes above this cutoff are referred to as true positive (real data) and false positives (permuted data). The fractions of true and false positive (TPR, FPR) genes, as well as the precision, recall and F1 measures that were calculated based on these TPR/FPR scores, are reported in plots D-II – D-IV. (**D-II**) Precision, recall and F1 values (x axis) across datasets (y axis) for a cutoff of $R^2 = 0.05$, using the joint model. The plot indicates the validity of the model in both healthy subjects and during infection (including sepsis). **(D-III**) Comparison of TPR and FPR. Each dot represents a single cohort dataset and a specific cutoff ($R^2 = 0.01, 0.05, 0.1, 0.5$). TPR values are consistently higher than the FPR values in different cutoffs. (**D-IV**) The tradeoff between TPR and FPR across the six cohorts and four R^2 cutoffs, using the joint model. We observe that both the FPR and TPR are consistent between the SI-only model and the R-only model, suggesting a similar accuracy of both programs across the various biological contexts.

(**E**) Evaluation of gene weights: analysis of inter-gene variation within individuals that is explained by the predefined gene weights of programs R and SI. (**E-I**) Examples. Box plots for the regression's R^2 using the SI/IM1 programs across all sepsis individuals (PBMCs, left) and for the regression's R^2 using the R/T programs across all individuals with S. aureus infection (blood samples, right). The plots present the analysis of real data and permuted data (x axis). The horizontal red lines indicate empirical p = 0.05 level. The fraction of individuals with empirical p <0.05 in real data (true positive rate, TPR) and

permuted data (false positive rate, FPR) is specified. Given that the R^2 presents the percentage of inter-gene variation that is explained by the predefined gene weights, the TPR and FRP, as well as the AUC that is calculated from these rates, indicate the ability of gene weights to predict inter-gene variation. (**E-II**) The percentage of individuals with empirical p <0.05 in real data (TPR) is reported for each dataset (column 1). As exemplified in panel E-I, the analysis allows estimation of false positive rate (FPR) and true positive rate (TPR) of predicted individuals for each R^2 cutoff, thereby allowing assessment of AUC (calculated across various R^2 cutoffs) in each dataset (column 2). In all datasets and all programs, we observe good performance (AUC >0.95) in detection of real individuals.

(**F**) R and SI levels are linked to a different inflammatory plasma state. (**F-I**) It was originally demonstrated that the resistance (R) program is engaged to eliminate the pathogen [1]. Particularly, it was shown that the level of resistance in lungs is tightly correlated with the viral burden, the mRNA levels of interferon genes and the mRNA levels of Cxcl10 and Cxcl11 in murine lungs [1]. Shown are the Pearson's correlation coefficients between R levels and infection parameters (y axis), calculated in different time points after *in vivo* IAV infection (x axis). Correlations between R levels and each infection parameter (in each time point) were calculated across 33 CC mouse strains. Shown are correlations of R levels with five parameters of the infection response (color coded). (**F-II**) It was originally demonstrated that the SI levels vary significantly between obese subjects and are tightly correlated with the percentage of lymphocytes in blood, as well as the IL6, IL18 and IL18BP proteins in plasma [2]. Shown are Pearson's correlation coefficients of SI levels with each of these four parameters. Correlations were calculated from blood parameters across obese human subjects. Data is shown in four distinct groups of subjects (color coded). Plots F-I, F-II were produced from data in Refs. [1],[2], respectively. Overall, our findings in sepsis patients (**Figure 1B,D**) are consistent with the original characterization of programs R and SI, as described in the original publications and demonstrated in plot F: IFNg, CXCL11 and CXCL10 in sepsis are mainly associated with program R, whereas IL6 and IL18bp in sepsis are mainly associated with program SI.

(**G**) SI and R levels in SIRS and sepsis. Data is shown for two datasets: a dataset with blood samples (**G-I;** GSE13904; dataset #22 in **Methods**) and a dataset with samples of isolated neutrophils (**G-II;** GSE123729; dataset #21 in **Methods**). Both datasets are detailed in **Table S2**. Each dataset consists of three groups: healthy controls, SIRS (in which negative blood culture was confirmed for all patients) and sepsis/septic shock (in which positive blood culture was confirmed for all patients). Thus, in each dataset, the SIRS and sepsis groups differ in the presence of pathogen (among other differences). **Left:** The levels of SI (x axis) and R (y axis) across individuals (dots) from each cohort. **Right:** Box plots for the SI and R levels in each individual group from each cohort. The plots show that SIRS is linked to a significant increase in SI levels but no increase in R levels. In sepsis, the results were similar to SIRS with a slight elevation of R levels. These results support the general role of SI in any type of inflammation (with or without pathogen) and the specific role of program R in exposure to pathogen, consistent with the notion that R and SI are resistance and systemic inflammation programs, respectively. Indicated are t test p-values; in G-II, due to the small dataset we also calculated empirical p-values using permutation tests (reported in parentheses).

Supp. Figure 2. R and SI levels during moderate infection and sepsis, related to Figure 2.

(**A**) Differential R and SI levels (disease versus controls, t-test statistics) across moderate infections (green), sepsis (yellow) and autoimmune disease (purple). The plot indicates that the R/SI balance in autoimmune disease is similar to the balance in moderate infections and is distinct from the balance in sepsis. Sources: datasets #1-#5 (sepsis), #6-#10 (moderate infections), #11 (autoimmunity) in **Methods**.

(**B**) Accounting for age and gender. (**B-I,II**) Differential R and SI levels (disease versus controls) across cohorts (dots). Presented are all cohorts from **Figure 2C** in which annotation of males and females is available (B-I) or annotation of age is available (B-II). B-I: Females and males were analyzed separately and are presented by different shapes. Differential levels were calculated using standard t-test statistics. B-II: Analysis of each cohort was applied either with covariates (square) or without covariates (circle). Covariates included are age and gender. For septic shock I and II only the age covariate was available. Differential SI/R levels were calculated using the coefficient of the disease term in a linear regression

model where R (or SI) is the dependent variable, disease is the independent variable, and age/gender are used as additional covariates.

(**C-E**) Temporal patterns of R and SI levels during moderate infection and sepsis. (**C**) R and SI levels across time points during moderate infections. Included are four time-series gene expression datasets (datasets #13-#15 in **Methods**); see additional time series datasets in **Figure 2E** and in panels D-E. Error bars: 95% confidence intervals. p.i., post infection; p.s., post symptoms. (**D**,**E**) Temporal patterns of R and SI levels in a murine sepsis model: *in vivo* LPS stimulation. Data from dataset #16 in **Methods** (Takahama et al. 2024). (**D**) R and SI levels calculated in murine PBMC following *in vivo* LPS stimulation. D-**I**: Error bars: 95% confidence intervals. D-**II**: Scatter plot visualization. Each circle is an individual mouse and time points are color coded. (**E**) R and SI levels calculated in 13 different tissues following *in vivo* LPS stimulation. E-**I**: heat map for the temporal response of each tissue. Time points and tissues are indicated on columns and rows, respectively. E-**II**: Scatter plot visualization, demonstrating the dynamic response in bone marrow and spleen. Each circle is an individual mouse and time points are color coded. Abbreviations are as in Takahama et al. 2024.

Supp. Figure 3. Molecular states of SI and R in sepsis patients, related to Figure 2.

Data from scRNA-seq profiling (dataset #17 in **Methods**), including 19 healthy controls, 10 urinary tract infection (UTI) patients showing clear symptoms of sepsis - i.,e., a clear and persistent organ dysfunction (denoted 'URO' in the original publication, Reyes, M. et al. 2020), and 10 UTI patients with moderate infection: a clear leukocytosis but no organ dysfunction (denoted 'Leuk-UTI' in the original publication, Reyes, M. et al. 2020). The analysis is focused on the subpopulation of monocytes (MS1-MS4), T cells (TS1, TS2), B cells (BS1,BS2) and NK cells (NS1,NS2).

(**A**) Demonstration of the R and SI levels in single monocytes. A two-dimensional space in which each gene (a dot) is located in accordance to its correlation with SI levels (*x* axis) and R levels (*y* axis) in PBMCs across individuals of the FUSE cohort. In each panel, the map is colored by the measured expression levels in a single monocyte cell. The blue/red scale refers to low/high gene expression levels (with smoothing). For each cell, the R and SI levels were calculated based on the gradients along the *x* and *y* axes, respectively; the R and SI levels of each cell are indicated on top (see calculation of R/SI levels in **Methods**). As demonstrated here, high (low) SI levels reflect increasing (decreasing) gradients along the *x* axis. Similarly, high (low) R levels reflect increasing (decreasing) gradients along the *y* axis.

(**B**) A significant molecular R and SI response of monocytes during the course of moderate infection and sepsis. For each individual (a dot), the plot presents its molecular responses (y axis). Responses are reported for both R and SI in each monocyte subpopulation (x axis). Sepsis and moderate UTI-infections are shown in two separate plots. For a large fraction of individuals, there is a significant response ($p<0.05$, above the horizontal line).

(**C**) Distributions of SI levels (top) and R levels (bottom) across single cells of all UTI-sepsis patients (yellow) and all UTI no sepsis (green). Distributions are shown for T cells, B cells, monocytes and NK cells (left to right). P-values for the difference between the sepsis and no-sepsis distributions are indicated on top. The resulting P-value of monocytes is indicated in **Figure 2F-II**.

(**D**,**E**,**F**) Results are shown as in plot B but for T cells, B cells and NK cells, respectively.

Supp. Figure 4. The relations of R and SI with signatures of sepsis, related to Figure 3.

(**A**) Plots demonstrating that the findings in **Figure 3AB** are reproduced in both females and males (**A-I**) and when adding the age and gender as covariates (**A-II**).

(**B**) A reduction in R-to-SI balance following exposure to septic plasma. In this experiment (Khaenam et al. 2014; dataset #19 in **Methods**), plasma samples were derived from several sepsis patients ('septic plasma') and several healthy subjects ('healthy plasma'). Next, cells from healthy patients were stimulated with each of the plasma samples; both granulocytes, PBMCs and DCs were stimulated. Finally, transcriptomes were generated for each cell type after stimulation with the plasma of each individual subject. Presented are scatter plots of SI and R levels in granulocytes, PBMCs and DCs, for stimulation with septic plasma or healthy plasma (color coded; septic plasma from S. Pneumoniae is

indicated with a different color). Each dot presents R/SI levels following stimulation with the plasma of a different individual subject. Bottom: *t*-test *p* values for the differences between healthy and septic plasma in either R levels, SI levels, or the R/SI-balance score. Overall, the impaired R/SI balance is observed in both DCs, granulocytes and PBMCs following the exposure to septic plasma.

(**C,D**) Transcriptional signatures of sepsis. The findings in **Figure 3C** are reproduced in both females and males (**C-I**) and when adding the age and gender as covariates (**C-II**). Plot D provides examples of specific genes, demonstrated as in **Figure 3E**.

Supp. Figure 5. Clinical implications of the R/SI framework, related to Figures 4 and 6.

(**A-D**) **R and SI are informative for sepsis pathology in the PROVIDE cohort**.

(**A**) The plasma signature of sepsis reflects the impaired R/SI balance. Plot is shown as in **Figure 3B** but using the PROVIDE data (dataset #18 in **Methods**). In general, associations of plasma proteins with R and SI that were detected in the FUSE cohort (**Figure 3B**) were also detected in PROVIDE.

(**B**) Validation of FGF-23 as a marker of the R/SI-balance score. Based on the FUSE cohort (**Table S3**), FGF-23 is the best protein marker of the R/SI balance score. The scatter plot demonstrates that it is also a marker of the R/SI-balance score in the PROVIDE cohort.

(**C-D**) Relations of R and SI with clinical parameters across patients in the PROVIDE cohort. For each clinical parameter, we calculated linear regression where the clinical parameter is the dependent variable, and the SI (or R) is the independent variable. (**C**) For each clinical parameter (a row), presented is the $\log_{10}$ p-value for the SI (or R) term. The log10 p-value is signed: a positive/negative sign for a positive/negative coefficient of the term. For $p<0.05$, the p-value is reported; for $p>0.05$, the entry is empty and white. Grey entry: p-value was not calculated (too few data points). Regressions were calculated either with females only (column 2), males only (column 3), females and males together without any covariate (column 4), and females and males with age as covariate (column 5). The pathologies in lines 1-8 are known to be higher in sepsis, and the pathologies in lines 9-10 (mHLA-DR and %lymphocytes) are known to have reduced values in sepsis; this is consistent with the opposite associations of these pathologies with R and SI levels. (**D**) Relations of clinical parameters with R and SI levels. (**D-I**) A scatter plot for the relations of each clinical parameter (a dot) with SI and R (x and y axis, respectively), shown for the regression of females and males together without covariates. Presented is the signed $\log_{10}$ p-value for the SI and R terms (a positive/negative sign for a positive/negative coefficient of the term). The analysis was conducted twice: using all sepsis individuals of the PROVIDE cohort (blue, *n*=223) and when excluding the high- -SI patients (peach, *n*=169). (**D-II**) Relations with disease severity. The bar plot indicates the relations of each parameter (y axis) with sepsis severity (using the SOFA score). The x axis presents the percentage of variance explained for regressions that were calculated with females and males together when using either all individuals or only low-SI individuals (color coded). The plots present comparison of the R/SI balance score to known inflammatory markers, as well as a separate regressions of R levels and SI levels. Low-SI individuals are all PROVIDE individuals excluding the high-SI endotype (*n*=169). In plots C-D, the analysis indicates significant associations of both R and SI, and further shows that R and SI present opposite directions of associations: in all cases, pathophysiological parameters are associated with either high SI, low R, or both.

(**E**) The R/SI balance is informative for sepsis severity in the FUSE cohort**.** The FUSE patients were grouped into severe and non-severe sepsis according to the clinical manifestation of disease (the qSOFA score, **Methods**). The plots present a comparison of severe (n=29) versus non-severe (n=94) sepsis in this cohort, using R and SI levels in PBMCs. Presented are a scatter plot (**E-I**) and distributions (**E-II**) of severe and non-severe sepsis patients, revealing lower R/SI-balance scores in severe sepsis compared to non-severe sepsis ($P<0.006$, t test). (**E-III,IV**) The performance of the R/SI-balance score as a classifier of sepsis severity. (**E-III**) Shown are ROC curves presenting the predictive performance of the R/SI-balance score (AUC = 0.75) and a currently established marker (ferritin, AUC = 0.54) for the

prediction of severe versus non-severe sepsis. We observed better performance of the R/SI-balance score in PBMCs throughout the entire ROC range compared to ferritin. Performing this analysis with age correction did not affect the results, although the p-values were less significant ($P < 0.01$ with age covariate in the linear regression). As shown in **E-IV**, neither an R-only score nor an SI-only score performed as well as the R/SI-balance score for stratifying disease severity.

(**F**) Modulation of the sepsis-related cell state in monocytes. R and SI levels were evaluated using transcription profiles of monocytes following different treatments (two repeats for each treatment). Dashed lines: 1.5 standard deviations, based on controls. Confidence intervals of R/SI levels were calculated by bootstrapping, using resampling of 50% of the genes. Abbreviations: DC – Diphenyleneiodonium chloride. For instance, the plot suggests that IFNg leads to increased R (but not SI) levels – that is, a pro-R treatment.

(**G**) An added value of the R/SI-based endotypes within each endotype of a previous classification. Comparison between a previously described classification (columns) and the new R/SI-based classification (rows) in the PROVIDE cohort. For each combination of current and new classes, indicated is the overall mortality rate (top, in bold), as well as the number of deceased patients out of the total number of patients (bottom). Included are only patients that were classified into one of three groups by the current and previous classifications.

Supp. Figure 6. Functional characterization of the impaired R/SI-balance state, related to Figure 4.

(**A**) The R/SI-balance mRNA markers show consistent associations in monocytes, PBMCs and blood samples of sepsis patients. For the selected 300 markers of good R/SI-balance scores and the selected 300 markers of impaired R/SI-balance scores (columns), presented are the correlation of each mRNA marker with the R/SI-balance score (color-coded), either in PBMCs, monocytes or whole blood (rows). (**B**) An unbiased functional characterization of the R/SI balance. We used an enrichment test to evaluate the overrepresentation of each functional class in the 300 markers from A (hyper-geometric test, $-\log_{10}$ FDR *q*-values; right/left directions indicate over-representation of gene sets in markers of the good/impaired-balance score). Consistent with the current knowledge about sepsis, the impaired R/SI-balance is marked by downregulation of gene expression functions and upregulation of extracellular matrix (ECM) functions – for instance, downregulation of expression-related functions such as Pol II transcription and mRNA splicing (*FDR* $q<10^{-11}, 10^{-12}$, respectively) and upregulation of ECM-related functions such as ECM organization and elastic fiber formation genes (FDR $q<10^{-11}$, 10^{-4}, respectively). The impaired R/SI balance also displays phenotypic characteristics of reduced proliferation and stemness: it is enriched with downregulation of cell cycle genes (e.g., FDR $q<10^{-12}$, 10^{-8} for 'cell cycle' and 'M-phase', respectively), downregulation of stem cells and hematopoiesis early progenitors, and inhibition of the activity of BMP2 in supporting embryonic development (FDR $q<10^{-19}$, $10^{-10}, 10^{-60}$, respectively). In agreement, genes that are typically repressed in embryonic cells (i.e., genes marked by the H3K27me3) are upregulated in the impaired R/SI balance state (FDR $q<10^{-5}$).

(**C-E**) An in-depth analysis of the role of cell proliferation for the R/SI balance in sepsis. It has been previously demonstrated that lymphopenia is strongly associated with sepsis, possibly due to low proliferation, or "quiescence", of immune cells. We therefore asked whether and how quiescence is related to the R/SI balance. We used a predefined "quiescence" gene set consisting of top expressed genes in quiescent (non-cycling) embryonic cells (**Methods**). (**C**) A scatter plot of SI and R levels in PBMCs (x and y axis, respectively) in each individual patient (a dot; including all sepsis patients from the FUSE cohort). The plot is colored by the averaged gene expression of the 85 quiescence genes. (**D**) **Top**: Correlation of each gene with SI (x axis) or R levels (y axis), which were calculated in PBMCs (left) or monocytes (right). Presented are all genes (gray) and quiescence genes (blue). **Bottom**: Scatter plots of SI and R levels (x and y axis, respectively) in each individual patient (a dot; including all sepsis patients from the FUSE cohort). Plots are colored by the gene expression of specific quiescence genes (indicated on top of each panel). SI and R levels were calculated in PBMCs (left panels) or monocytes (right panels). (**E**) Shown are correlations (color-coded) between each quiescence gene (column) and the R or SI levels (indicated on right). Correlations were calculated using transcription profiles in each cohort (rows). Abbreviations: SS-I/II – septic shock cohort I and II; cohorts are detailed in **Table S2**.

Overall, plots C-E indicate association of quiescence with low R and high SI. As the quiescence state of low R and high SI is highly prevalent in sepsis compared to moderate infections, lymphopenia is prevalent in sepsis.

Supplemental references

[1] Cohn, O. et al. (2022). Distinct gene programs underpinning disease tolerance and resistance in influenza virus infection. Cell Syst 13, 1002-1015.e9.

[2] Frishberg, A. et al. (2021). An integrative model of cardiometabolic traits identifies two types of metabolic syndrome. eLife 10, e61710.

Supp. Figure 1

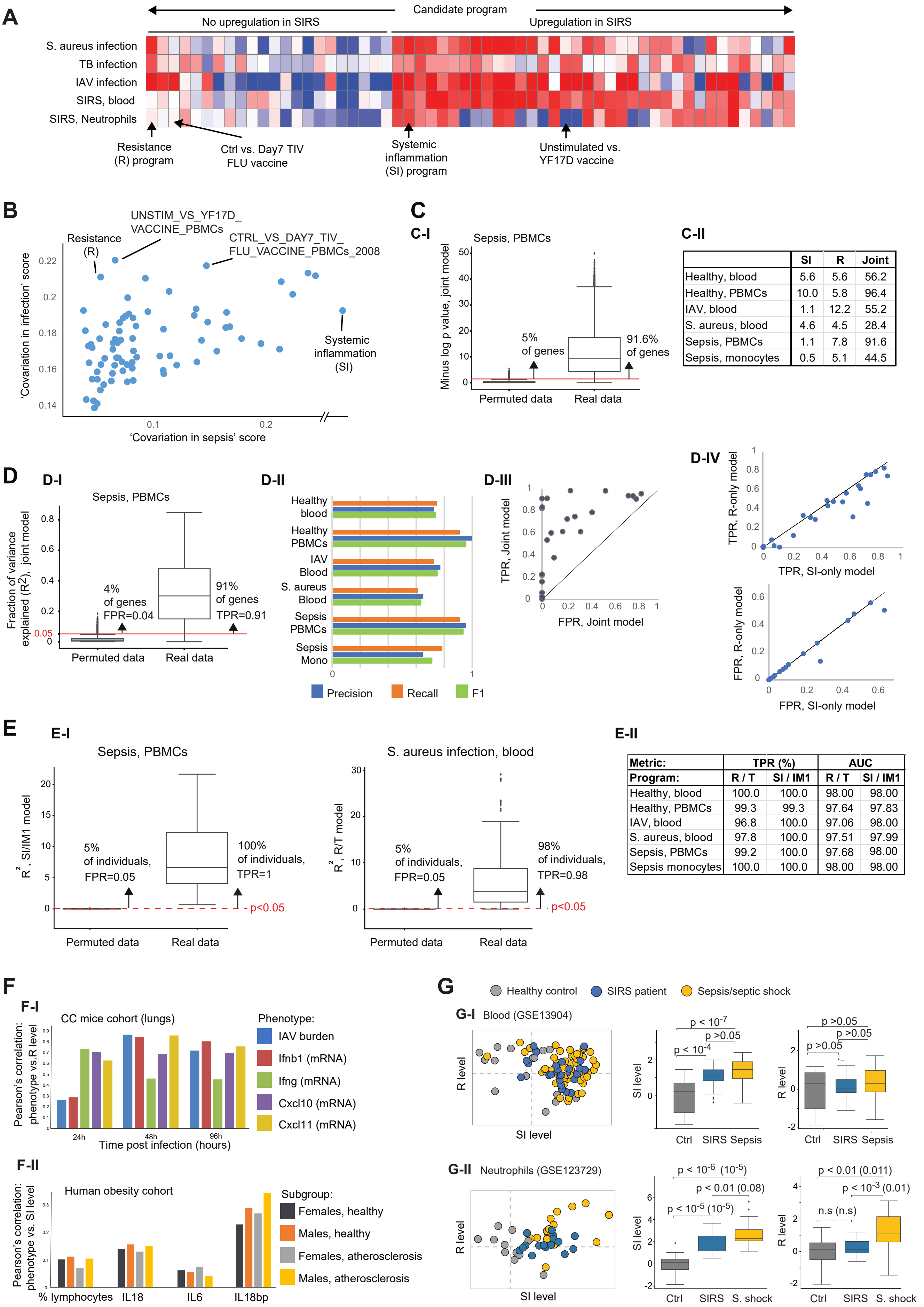

C-II

	SI	R	Joint
Healthy, blood	5.6	5.6	56.2
Healthy, PBMCs	10.0	5.8	96.4
IAV, blood	1.1	12.2	55.2
S. aureus, blood	4.6	4.5	28.4
Sepsis, PBMCs	1.1	7.8	91.6
Sepsis, monocytes	0.5	5.1	44.5

E-II

Metric:	TPR (%)		AUC	
Program:	R / T	SI / IM1	R / T	SI / IM1
Healthy, blood	100.0	100.0	98.00	98.00
Healthy, PBMCs	99.3	99.3	97.64	97.83
IAV, blood	96.8	100.0	97.06	98.00
S. aureus, blood	97.8	100.0	97.51	97.99
Sepsis, PBMCs	99.2	100.0	97.68	98.00
Sepsis monocytes	100.0	100.0	98.00	98.00

Supp. Figure 2

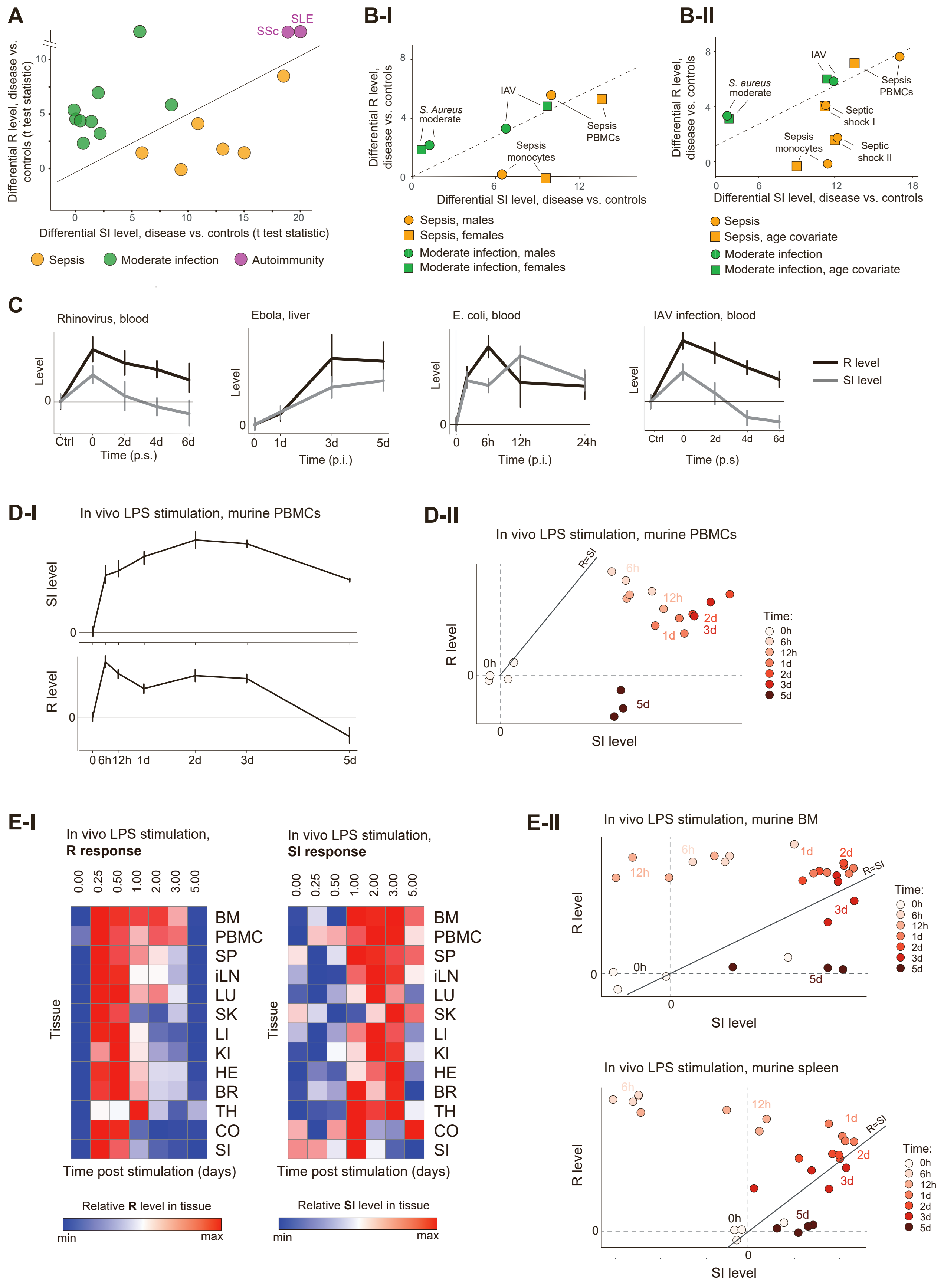

Supp. Figure 3

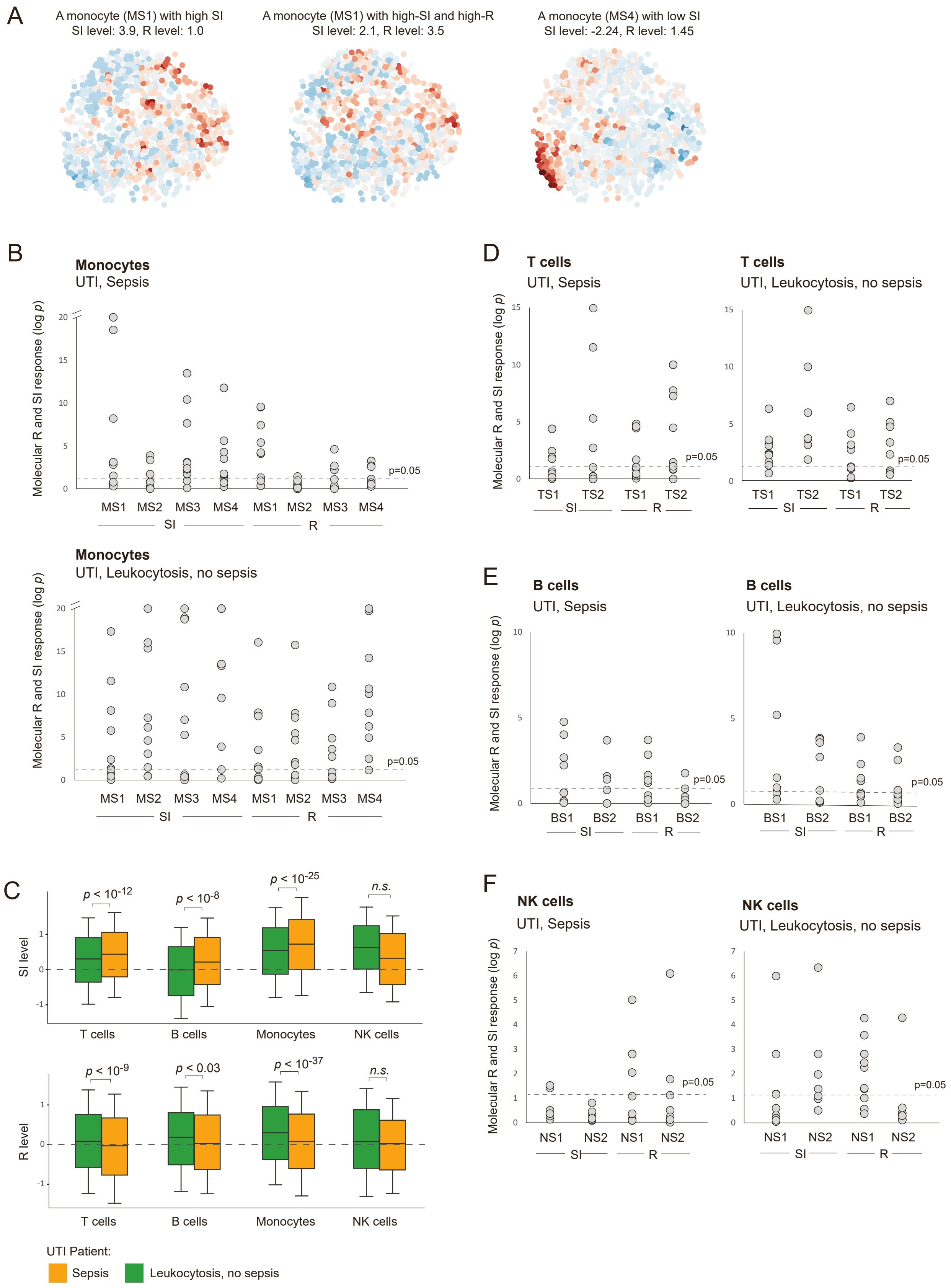

Supp. Figure 4

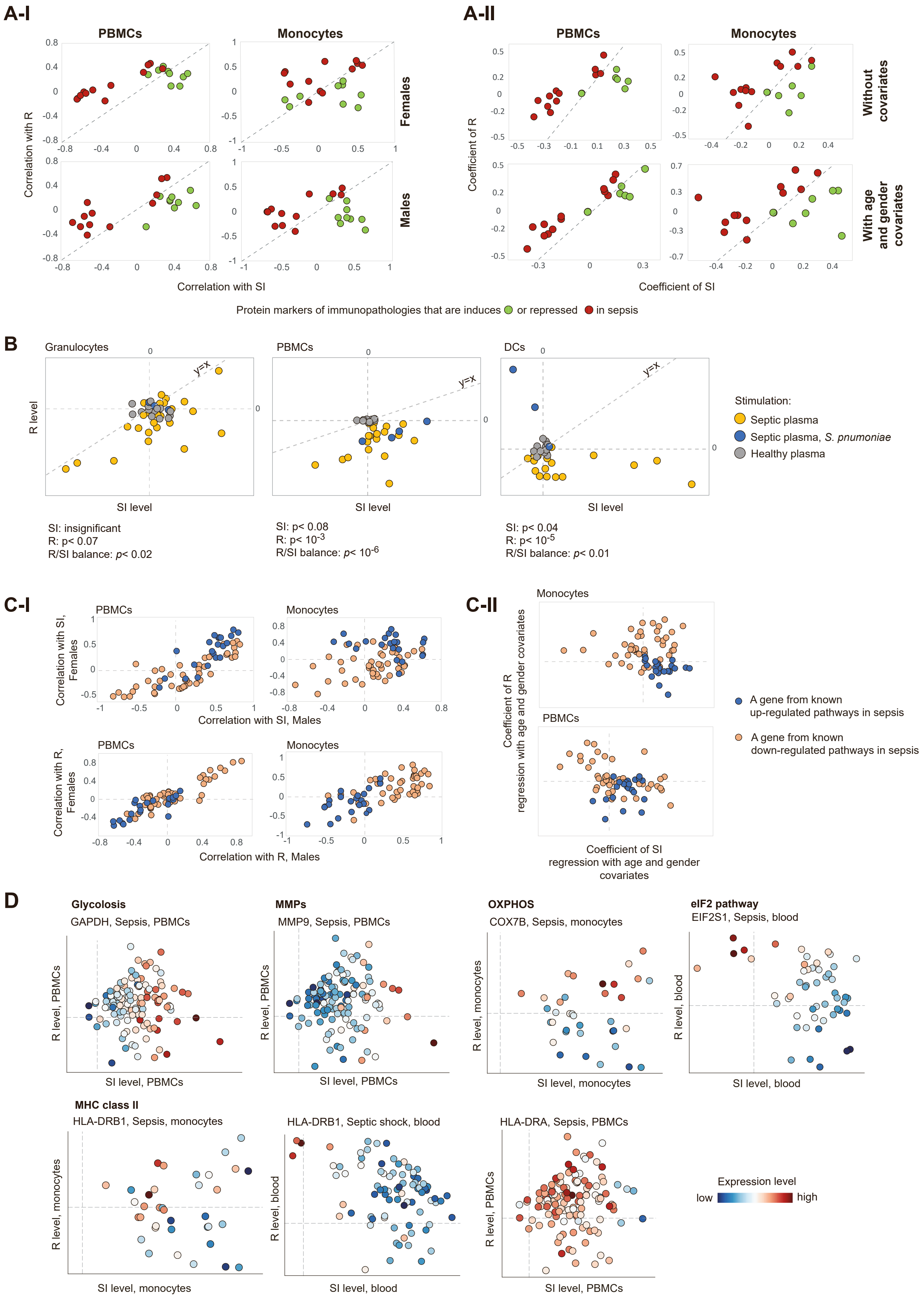

Supp. Figure 5

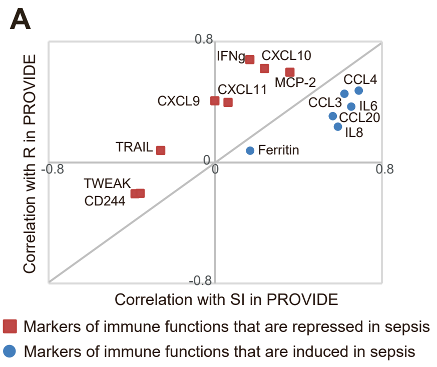

C

	Relation with SI (signed log p), PROVIDE				Relations with R (signed log p), PROVIDE			
	Females	Males	No covariate	Age covariate	Females	Males	No covariate	Age covariate
CRP		1.35	1.27	1.27				
NLR	3.5	2.4	5.3	5.26			-1.35	
%Neutrophils	2.0	2.9	4.34	4.26				
Hemodialysis					-2.10		-1.59	-1.70
Severity (SOFA)	3.3	5.27	8.13	9.00	-2.92	-1.84	-4.03	-4.97
Septic shock	4.4	0.70	3.67	3.79	-4.51		-3.18	-3.33
Lactate	2.0	3.04	4.63	4.45		-1.50	-2.18	-1.97
WBC	3.0	1.4	3.85	3.65	-1.71		-2.18	-1.92
%Lymphocytes	-3.6	-3.7	-6.84	-6.49	2.02	1.39	2.80	2.52
mHLA-DR		-1.3	-2.10	-2.01		1.22	1.34	

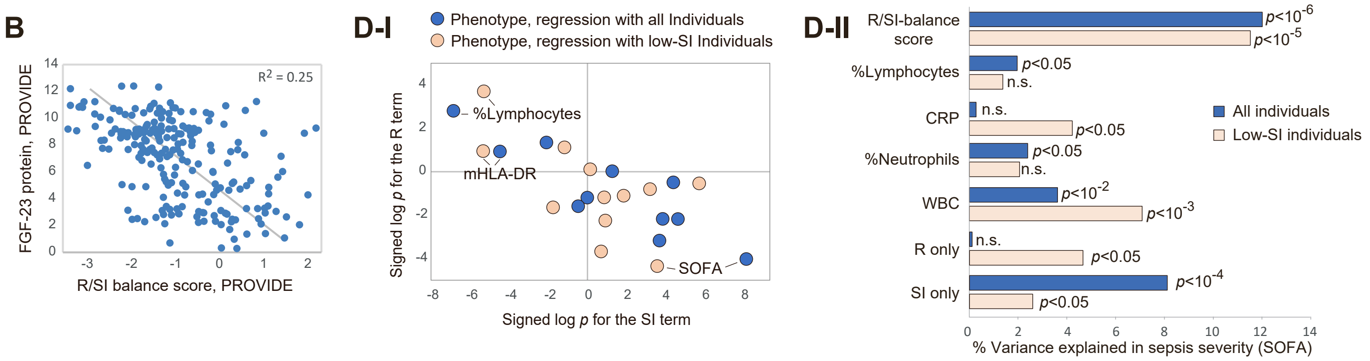

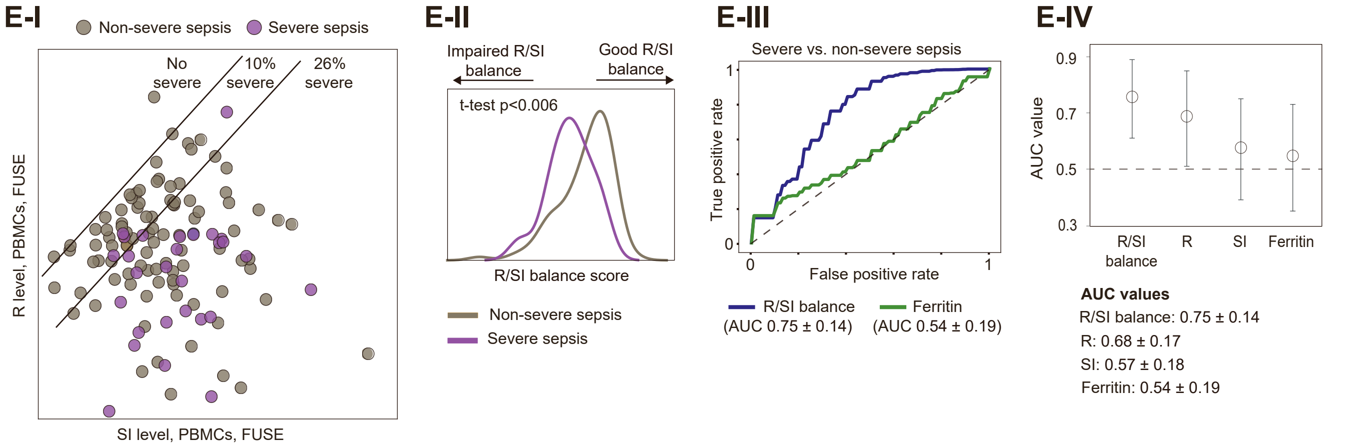

AUC values
R/SI balance: 0.75 ± 0.14
R: 0.68 ± 0.17
SI: 0.57 ± 0.18
Ferritin: 0.54 ± 0.19

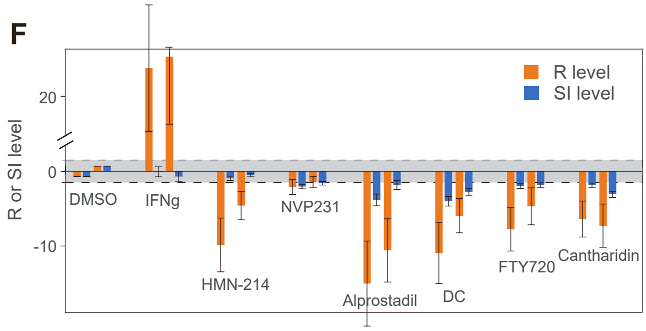

G

R/SI-based classification	Previous classification: MALS	Immunosuppression	Unclassified	All
SI-high	**92%** 12 of 13	**85%** 12 of 14	**80%** 4 of 5	**88%**
Severe R/SI imbalance	**81%** 22 of 27	**60%** 35 of 58	**42%** 18 of 43	**59%**
Moderate R/SI imbalance	**43%** 3 of 7	**50%** 7 of 14	**30%** 7 of 23	**39%**
All	**79%**	**63%**	**41%**	**59%**

Supp. Figure 6

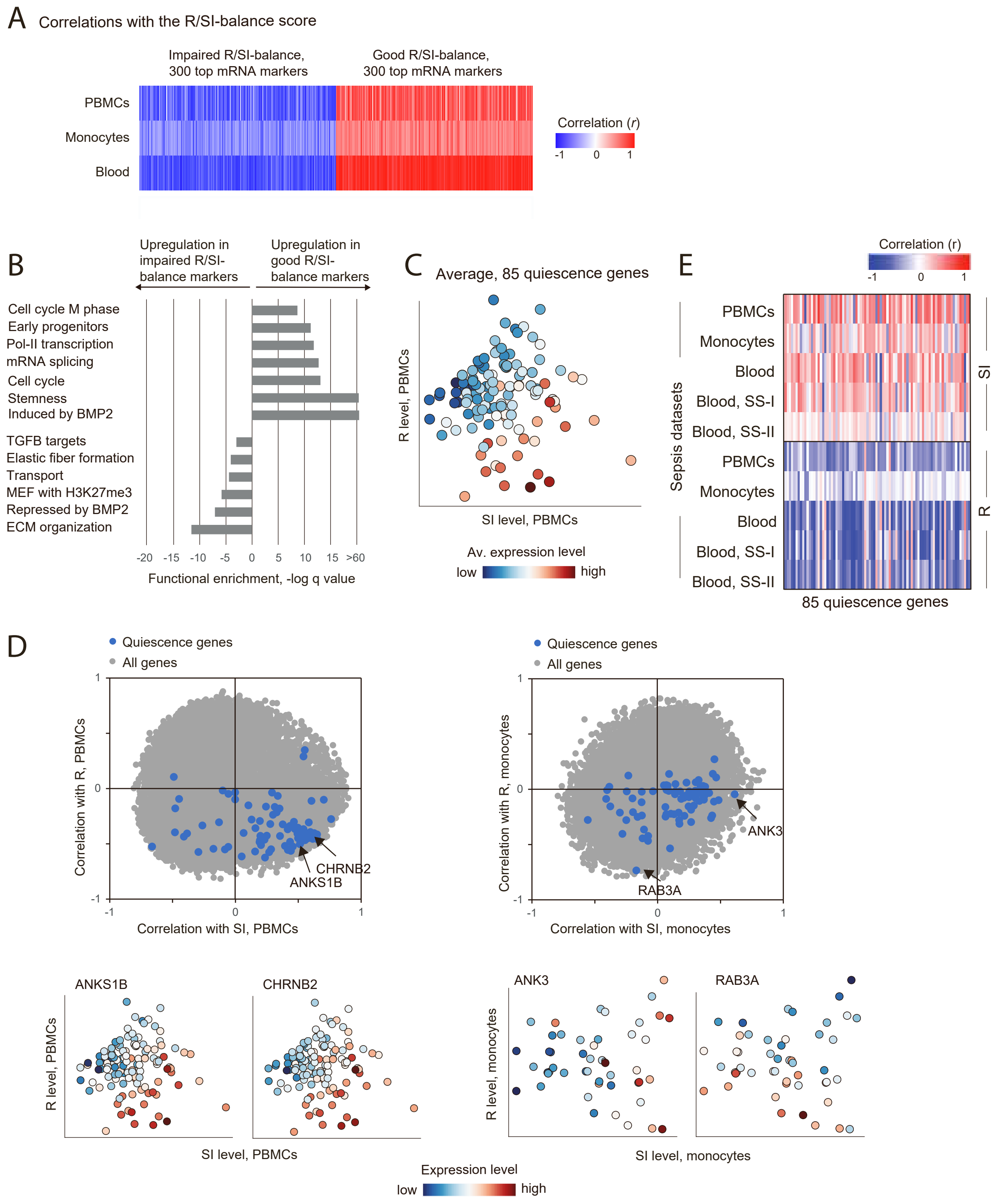

Supplement: Document S1. Figures S1–S6 [file mmc1.pdf]
